# Layered Structure and Complex Mechanochemistry Underlie Strength and Versatility in a Bacterial Adhesive

**DOI:** 10.1128/mBio.02359-17

**Published:** 2018-02-06

**Authors:** Mercedes Hernando-Pérez, Sima Setayeshgar, Yifeng Hou, Roger Temam, Yves V. Brun, Bogdan Dragnea, Cécile Berne

**Affiliations:** a Department of Chemistry, Indiana University, Bloomington, Indiana, USA; b Department of Physics, Indiana University, Bloomington, Indiana, USA; c Department of Mathematics, Indiana University, Bloomington, Indiana, USA; d Department of Biology, Indiana University, Bloomington, Indiana, USA; University of Michigan—Ann Arbor

**Keywords:** atomic force microscopy, bacterial adhesion, bioadhesive, *Caulobacter crescentus*, holdfast

## Abstract

While designing synthetic adhesives that perform in aqueous environments has proven challenging, microorganisms commonly produce bioadhesives that efficiently attach to a variety of substrates, including wet surfaces. The aquatic bacterium *Caulobacter crescentus* uses a discrete polysaccharide complex, the holdfast, to strongly attach to surfaces and resist flow. The holdfast is extremely versatile and has impressive adhesive strength. Here, we used atomic force microscopy in conjunction with superresolution microscopy and enzymatic assays to unravel the complex structure of the holdfast and to characterize its chemical constituents and their role in adhesion. Our data support a model whereby the holdfast is a heterogeneous material organized as two layers: a stiffer nanoscopic core layer wrapped into a sparse, far-reaching, flexible brush layer. Moreover, we found that the elastic response of the holdfast evolves after surface contact from initially heterogeneous to more homogeneous. From a composition point of view, besides *N*-acetyl-*d*-glucosamine (NAG), the only component that had been identified to date, our data show that the holdfast contains peptides and DNA. We hypothesize that, while polypeptides are the most important components for adhesive force, the presence of DNA mainly impacts the brush layer and the strength of initial adhesion, with NAG playing a primarily structural role within the core. The unanticipated complexity of both the structure and composition of the holdfast likely underlies its versatility as a wet adhesive and its distinctive strength. Continued improvements in understanding of the mechanochemistry of this bioadhesive could provide new insights into how bacteria attach to surfaces and could inform the development of new adhesives.

## IMPORTANCE

There is an urgent need for strong, biocompatible bioadhesives that perform underwater. To strongly adhere to surfaces and resist flow underwater, the bacterium *Caulobacter crescentus* produces an adhesive called the holdfast, the mechanochemistry of which remains undefined. We show that the holdfast is a layered structure with a stiff core layer and a polymeric brush layer and consists of polysaccharides, polypeptides, and DNA. The DNA appears to play a role in the structure of the brush layer and initial adhesion, the peptides in adhesive strength, and the polysaccharides in the structure of the core. The complex, multilayer organization and diverse chemistry described here underlie the distinctive adhesive properties of the holdfast and will provide important insights into the mechanisms of bacterial adhesion and bioadhesive applications.

## INTRODUCTION

The versatility and diversity of industrially produced adhesives pale in comparison to those of biological adhesives. Many organisms, from microscopic bacteria to larger vertebrates, are able to attach to surfaces, using strategies based on the production of elaborate adhesive biomolecules or on complex physical structures. For example, mussels and barnacles produce a multiprotein complex that acts as a wet adhesive to attach to various surfaces (1, 2). On the other hand, the toe pads of geckos are composed of a hierarchical structure of lamella consisting of thousands of micron-sized setae. Each seta is made of hundreds of nanoscale spatulas that mediate strong attachment via van der Waals and capillary interactions (3). However, the widest diversity of bioadhesives is produced by microorganisms (4).

Bacteria have evolved bioadhesives to colonize most surfaces. Atomic force microscopy (AFM) has been used to obtain three-dimensional images of adhesins at nanoscale resolution and to study their physical and chemical properties, either as components of whole bacterial cells or as part of purified systems (5–8). Whereas single-cell force spectroscopy (SCFS), where AFM tips are functionalized with bacterial cells, enables researchers to quantify adhesive properties in different microorganisms (9–11), single-molecule force spectroscopy (SMFS) has been developed to unravel the material properties of purified bioadhesives in cell-free systems, at the single-molecule level (12, 13). Nanoindentation experiments, which focus on the approach of using a sample and its indentation by an AFM probe (extension curves), are used to characterize long-range interactions and mechanical properties such as sample viscoelasticity, stiffness, and the Young’s modulus (14–17). On the other hand, the study of retraction curves, when the AFM tip moves away from the sample, allows characterization of adhesion forces (8, 11).

Some of the best-studied bacterial adhesins are proteinaceous and include long polymeric structures, such as fimbriae and pili, and short nonfimbrial adhesins, all of which are directly anchored to the cell surface (18). Recent biophysical studies of bacterial protein adhesins, conducted by AFM, have led to models whereby the concerted actions of multiple relatively weak adhesion molecules (19–21), microdomain accumulation of adhesins at the cell-substrate interface (22), or conformation changes (23), can mediate strong bacterium-substrate interactions. Another emerging theme is that multidomain protein adhesins can switch among different functional domains to ensure adhesion to a variety of substrates (24–27).

**FIG 1:**
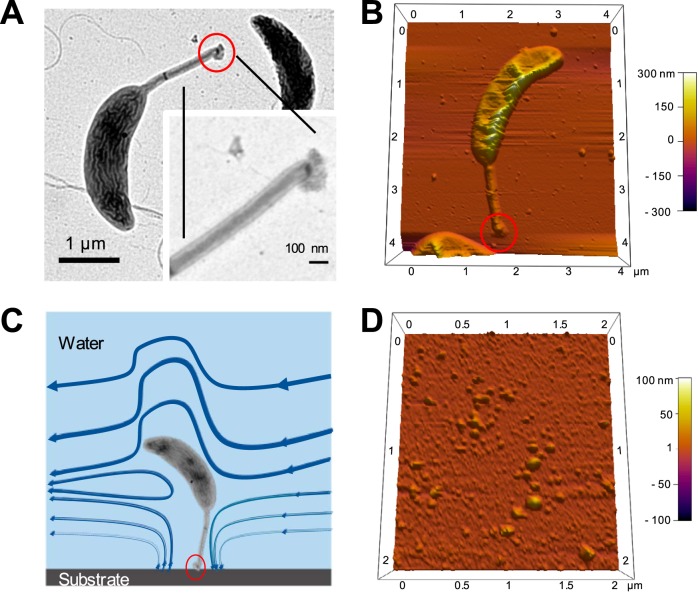
*Caulobacter crescentus.* (A and B) TEM (A) and AFM (B) images of a *C. crescentus* CB15 wild-type cell. (C) Schematic of *Caulobacter* attached to a surface under fluid flow. The holdfast size is in the order of tens of nanometer in diameter. The holdfast is circled in red. (D) AFM image of purified holdfasts attached to a mica surface in dH_2_O.

Many bacterial adhesives are polysaccharide based. While there is a large body of work on the properties of mechanochemistry at the single-molecule level of single polymeric polysaccharides (28, 29), only a few studies have investigated the bulk physicochemical properties of bacterial polysaccharide adhesives (30, 31). Unipolar polysaccharide adhesins are among the best-studied bacterial adhesives. This type of adhesin is produced at the bacterial cell pole in multiple genera of the *Alphaproteobacteria* (18, 32, 33). The holdfast, a nanoscopic adhesive produced by the oligotrophic freshwater bacterium *Caulobacter crescentus*, is the archetypical polarly localized adhesin (Fig. 1A and B). *Caulobacter* is commonly found in aquatic environments that are low in nutrients such as pristine lakes, tap water supplies, or distilled water tanks (34–36). The *Caulobacter* holdfast is remarkably versatile, e.g., is able to adhere to a variety of biotic and abiotic surfaces (18, 37), and exceptionally strong, with a tensile strength exceeding 68 N/mm^2^, one of the highest levels measured for both synthetic and biological adhesives (38). These properties make the holdfast a particularly interesting bioadhesive, holding potential for a variety of applications (39).

The holdfast adhesive is concentrated in a small, elastic structure (40) (<50 nm in height) located at the end of a long, thin extension of the cell envelope called the stalk (stalk dimensions, 80 nm by 1,500 nm) (Fig. 1A and B). In natural environments, the stalk places the cell body well within the flow and away from the stagnant substrate boundary layer, allowing improved access to nutrients under typically oligotrophic conditions (41). The tradeoff for this adhesion strategy, which improves access to nutrients, is that large drag forces on the cell body have to be balanced by an anchor with a nanoscopic footprint (Fig. 1C). Because of the differences in stress magnitude and type with respect to adhesives distributed over the whole cell surface, the holdfast might possess composition and structure distinctly different from those of previously studied distributed bioadhesives. Nevertheless, determinations of holdfast morphology and composition have been challenging, due to its high adhesiveness and insolubility and to the small amount (less than 10^−3^ µm^3^) produced by each bacterium.

Electron microscopy (EM) studies (36, 37, 42, 43) and AFM imaging data (40, 44, 45) suggest that the holdfast is amorphous. Newly secreted holdfasts appear to spread over the surface as a viscous fluid that subsequently hardens (44). Indeed, SMFS studies conducted on purified holdfasts showed that holdfast adhesion is time dependent and that adhesion force increases quickly over time (45). That study also suggested the presence of discrete, diffusible adhesins of an unknown nature that are responsible for the bulk of adhesion (45). However, until now, the only known component of the holdfast has been a polymer of *N*-acetyl-*d*-glucosamine (NAG) (42), which, by itself, cannot be responsible for the observed strong adhesion.

Here, we studied the elastic and adhesive properties of purified holdfast material, i.e., of holdfasts that are not connected to a stalk, which afford the possibility of conducting mechanochemistry studies without interference from cellular components (Fig. 1D). We show that holdfast adhesive properties are likely the result of a complex structural and compositional organization. Results from AFM nanoindentation experiments performed on substrate-supported holdfasts are consistent with the presence of a layered structure with a sparse and very flexible biopolymeric brush layer at the surface supported by a stiff, solid, elastic core. Results of enzymatic-treatment experiments suggest that peptides and DNA molecules are important for the integrity of the brush layer. The core seems to contain the bulk of NAG polymers. Finally, we present results of dynamic adhesion force spectroscopy experiments, which allow formulating potential roles for the different components. Thus, peptides are likely crucial for adhesion strength; NAG residues might provide structural connectivity; and the DNA is potentially involved in initiating adhesion. Following the lead in the development of bioinspired adhesives on the basis of mussel DOPA (3,4-dihydroxyphenylalanine) wet adhesives and the physical structures of the gecko seta (46–48), further mechanistic studies of complex adhesives such as the holdfast could inform the development of even more versatile adhesives inspired by nature.

## RESULTS AND DISCUSSION

### Mechanical characterization of the holdfast by AFM indentation and creep experiments.

**FIG 2:**
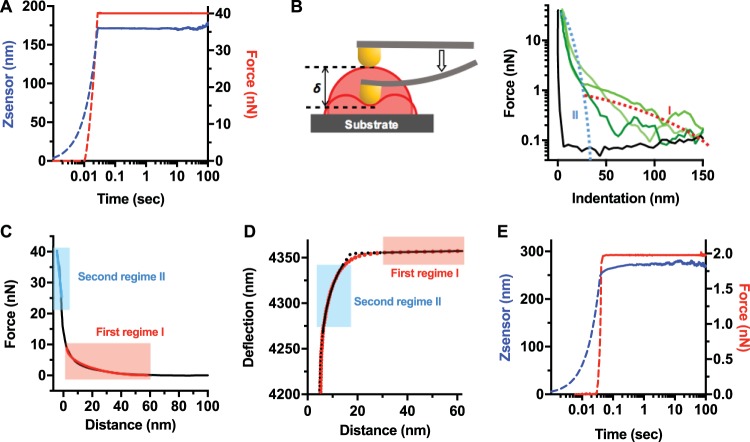
Holdfast response to AFM indentation and creep compliance experiments suggests a layered structure. (A) Representative creep compliance curve from a holdfast measured with a constant loading force of 40 nN and a dwell time of 120 s, to probe holdfast core (from 32 curves recorded during 3 independent experiments). In this experiment, the AFM tip indented the holdfast particle until the trigger force was reached (dotted curves). Once the probe reached the set trigger force (40 nN), the particle was kept at a constant force (red curve) for 120 s (solid curves). Particle deformation over time was recorded as changes in z-piezo extension (*Z-*sensor channel, blue curve). (B) (Left) Schematic illustrating the indentation (δ) of the AFM tip, deforming the holdfast, as described in the main text. (Right) Force-versus-indentation curves for the mica substrate (black curve) and three holdfast particles (each shade of green represents a different particle). Illustrations of regime I (red dotted line) and regime II (blue dotted line) are starkly different with respect to the magnitude and range of force opposing tip advancement. (C) A representative indentation curve (force versus distance) from a control, nontreated holdfast incubated for 16 h on the surface (from 310 curves recorded on 86 holdfasts during 10 independent experiments), showing separate fits to the brush layer model in the first regime (red line) and an effective, linear spring model in the second regime (blue line). (D) Simultaneous fits to brush layer and simple Hertz models in the first and second regimes, with fit parameters given by AFM tip deflection far from the sample. *d*_0_ = 4.2 × 10^−9^ m; holdfast height (*h*) = 42.5 × 10^−9^ m; holdfast surface (*Z*_0_) = 4.357 × 10^−6^ m; Young’s modulus of the holdfast core (*E*_bulk_) = 0.348 × 10^9^ Pa; brush layer thickness (*L*_0_) = 279 × 10^−9^ m; brush layer density (Γ) = 0.31 × 10^17^ m^−2^. The fits exclude a transition region at the surface of holdfast where neither a linear elastic model nor a brush layer model strictly hold. (E) Representative creep experiment curve performed on a holdfast brush layer (16 h after deposition on mica) with a constant force of 2 nN and a dwell time of 180 s (from 21 curves recorded during 2 independent experiments). First, the tip indents the holdfast particle until the trigger force is reached (dotted lines). Then, once the probe reaches the set trigger force, the particle is deformed at a constant force for 180 s (solid lines). The particle deformation over time is recorded as changes in the piezo extension *Z* (*Z-*sensor channel, blue curve) under a constant loading force (red curve).

The holdfast is believed to initially display fluid-like properties, rapidly spreading on the substrate upon contact within minutes (44). However, strong adhesion requires a viscoelastic or elastic-solid adhesive. To determine whether the holdfast behaves as a viscoelastic material and to obtain insight into the relaxation times, we performed creep compliance experiments (49) on holdfasts deposited on a freshly cleaved mica surface, 16 h before AFM measurements. In these experiments, the AFM probe, located at the end of a cantilever, applies a constant loading force on top of the holdfast. The deformation of holdfast in response to the set applied load as a function of time (creep) was measured (50). This method has been successfully used to determine the viscoelastic properties of single bacteria (51, 52). A potential time dependence of the holdfast deformation at a constant load would point to its being a viscoelastic material (53). The time dependence is easier to observe in creep experiments than in conventional indentation experiments because the latter require deconvolution of the tip motion, which is a time-dependent function of the contact area size. Figure 2A shows a representative creep compliance curve on a holdfast. During the first 0.02 s, the AFM tip pressed down on the holdfast until the selected loading force (40 nN) was reached. We found that, after rapidly applying the constant loading force of 40 nN, holdfasts did not exhibit measurable creep over a dwell time of 120 s (Fig. 2A). If deformation had been observed over a period of time, the material would have qualified as viscoelastic. Instead, cured holdfasts respond mechanically as an elastic solid, at least over a time scale of several minutes, to what amounts to being considerable stress for a biological material.

We then performed nanoindentation experiments on holdfasts attached on the surface, focusing on the approach AFM curves. We were able to observe two clearly different interaction regimes between the AFM tip and the holdfast (blue and red dotted curves in Fig. 2B). The first regime (regime I) was characterized by interaction forces of less than 1 nN and by a strikingly long range (up to 120 to 150 nm above the mica surface). The second regime (regime II) was characterized by a much stiffer response and started at around 25 to 40 nm above the mica surface. Note that the location of the interface between the two regimes coincides with what is conventionally considered to be holdfast height, as measured by AFM (see Fig. S1 in the supplemental material) (38, 45). What are the origins of these two regimes, so different in terms of force magnitude and range?

10.1128/mBio.02359-17.2 FIG S1  Holdfast height distribution. (A) The heights of shed holdfasts in solution were measured by AFM (37 holdfasts, 16-h incubation, 2 independent experiments). (B) Schematic of holdfast, as a hemisphere of 40 nm (core only). Download FIG S1, TIF file, 14.1 MB. Copyright © 2018 Hernando-Pérez et al. 2018 Hernando-Pérez et al. This content is distributed under the terms of the Creative Commons Attribution 4.0 International license.

**FIG 3:**
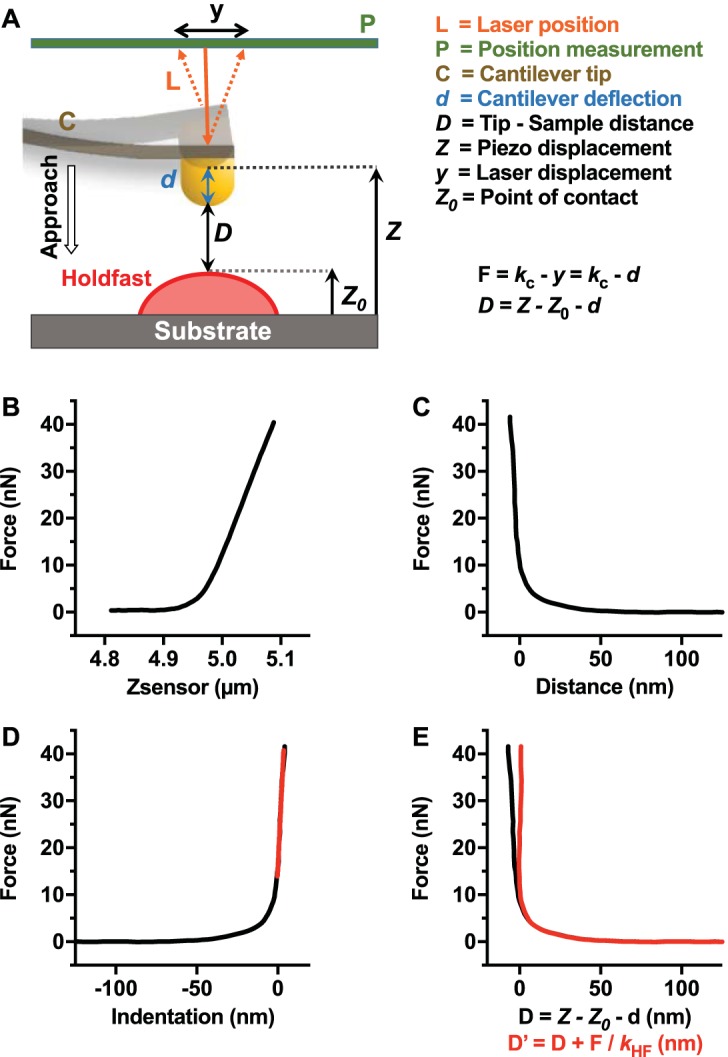
AFM indentation experiment analysis. (A) Schematic representation of the fundamental tip-sample interaction forces of a single force-distance curve. (B) Representative curve illustrating *F* versus *Z* recorded during indentation experiments on holdfast (from 310 recorded curves). (C) Conversion of data presented in panel B to *F* versus *D* curve (where *D* is the relative distance between tip and holdfast *D = Z – Z*_0_ *– d*). (D) Force-versus-indentation curve. (E) Comparison between *D* and *D*′ = D + *F*/*k*_HF_ (red) where *D*′ is the real gap distance between tip and holdfast.

We first focused on the characterization of regime II (blue in Fig. 2B and C), defined by steep slopes in the force-versus-indentation curves corresponding to deformations of the innermost layer, or core, of the holdfast. Since creep compliance experiments revealed that the holdfast core behavior was consistent with its being a solid, elastic body (Fig. 2A), it was possible to obtain an effective spring constant of the holdfast core from the slope of the approximately linear part of indentation curves (Fig. 2C and 3). For indentation depths that were on the order of 5% to 15% of the height of the holdfast core, the root mean square deviation from the linear fit slopes was less than 2%. We observed a broad distribution of apparent stiffness values of 9.0 ± 12.6 N/m (median ± standard deviation). The wide breadth of data distribution suggests significant heterogeneity at both the intra- and interparticle levels (Fig. S2A). We note that the measured stiffness is extremely high for a bioadhesive and is 1 to 2 orders of magnitude higher than that measured on exopolysaccharides (EPS) or lipopolysaccharides (LPS) present on the surface of bacteria (54).

10.1128/mBio.02359-17.3 FIG S2  Core stiffness of holdfast at 16 h (A) and 64 h (B). A total of 20 different random holdfasts are shown (from the 86 and 59 total holdfasts measured during at least 8 independent experiments), represented by different colors, with each circle giving the effective spring constant from a single nanoindentation measurement. Column bars stand for means ± standard deviations. Download FIG S2, TIF file, 14.1 MB. Copyright © 2018 Hernando-Pérez et al. 2018 Hernando-Pérez et al. This content is distributed under the terms of the Creative Commons Attribution 4.0 International license.

Since the tip/sample area increases while indenting takes place, the dependence of force on indentation is nonlinear. This nonlinearity is described by the Hertz model for small indentations (55) (Fig. 2D; see also Text S1 in the supplemental material). A fit using this model to analyze force versus indentation data in regime II allowed estimating the average Young’s modulus, *E*, of the core material at 0.37 ± 0.18 GPa (Table 1). These values are comparable to those determined for amyloid fibers (56), which are involved in adhesion of different bacteria (57), and for mussel byssal threads (58) but are 1 and 2 orders of magnitude higher than those determined for the EPS from *Staphylococcus epidermidis* (31) and *Pseudomonas aeruginosa* (59), respectively.

10.1128/mBio.02359-17.1 TEXT S1  Multiregime description of holdfast material properties. Download TEXT S1, PDF file, 0.6 MB. Copyright © 2018 Hernando-Pérez et al. 2018 Hernando-Pérez et al. This content is distributed under the terms of the Creative Commons Attribution 4.0 International license.

**TABLE 1:** Summary of estimated equilibrium length, density of biopolymer, stiffness, and elastic modulus of holdfast under conditions of maturation and enzyme treatments

Sample parameter	Value(s) (median ± SE)^a^									
	16 h (control)			64 h			16 h			
	Separate fits	Simultaneous fits		Separate fits	Simultaneous fits		+ 10 mM NaCl	Proteinase K	DNase I	Lysozyme
		Transition excluded	Transition included		Transition excluded	Transition included				
*n*	310	59	51	298	59	54	64	87	130	127
Spring constant (N/m)	9.0 ± 0.7	NA^b^	NA	7.1 ± 0.5	NA	NA	18.4 ± 1.9	28.3 ± 3.0	5.9 ± 1.3	5.7 ± 1.90
Young’s modulus (GPa)	NA	0.37 ± 0.18	0.37 ± 0.15	NA	0.36 ± 0.12	0.36 ± 0.08	NA	NA	NA	NA
Polymer length *L*_0_ (nm)	89.8 ± 5.2	213 ± 28	34.6 ± 4.9	65.4 ± 4.3	153 ± 9.8	28.5 ± 3.3	39.49 ± 2.5	49.9 ± 3.0	34.3 ± 2.0	71.0 ± 4.3
Polymer density Γ (molecules/m^2^) × 10^17^	0.84 ± 0.08	0.36 ± 0.02	1.12 ± 0.06	1.24 ± 0.06	0.55 ± 0.04	1.24 ± 0.08	2.1 ± 0.11	1.64 ± 0.19	2.47 ± 0.19	1.17 ± 0.17

a Separate-fit data were calculated based on the separate fits to the linear portion of the second regime (for the spring constant values) and the brush layer model fit of the first regime (for the polymer length and density values), as described in the text. Simultaneous-fit data were calculated based on the simultaneous fits of both regimes, as described in the main text and Text S1 in the supplemental material.

b NA, nonavailable.

Several observations can be made at this point. First, previous work (60) analyzed fluctuations in stalk angle for a pair of whole cells attached to a single holdfast, measuring an effective torsional spring constant from which an elastic modulus, *E* = ≈2.5 × 10^−5^ GPa, is determined. This value is approximately 4 orders of magnitude lower than that obtained by direct compression measurements of purified holdfast reported here. A possible explanation for this discrepancy is that the torsional spring constant obtained from whole-cell measurements reflects the shear modulus of the connection between the holdfast and the stalk, in addition to that of the holdfast itself. Second, we note that the simple form of the Hertz model assumes that the sample is homogeneous, isotropic, and infinitely thick. Real biological samples deviate from these idealized conditions. Holdfast thickness, in particular, is only ~40 nm (Fig. S1). Deviations from the Hertz model are expected when the extent of the stress field spans a significant portion of the sample thickness and when the substrate and the sample have very different compression moduli (61). In this regard, the estimate obtained for the Young’s modulus for a holdfast, within the Hertz model approximation, is much less than that for muscovite mica (100 to 200 GPa). While the deformation in response to indentation force is consequently expected to be dependent on the thickness, the force-indentation response curves do converge at small indentations and thicknesses larger than 20 nm (Fig. S3). Thus, while we have included finite thickness corrections to the simple Hertz model (see Text S1 in the supplemental material) (62, 63), the results of determinations using fits with and without finite thickness corrections have been consistent (to within a factor of ~2), demonstrating that under our experimental conditions, the substrate does not impact the order of magnitude of the measured holdfast stiffness.

10.1128/mBio.02359-17.4 FIG S3  Finite element simulation of the deformation of a thin holdfast layer with Young’s modulus given by 0.2 GPa for thicknesses of *h* = 120 nm (A), 60 nm (B), and 12 nm (C). The AFM tip is a cylindrical indenter with a radius of 13 nm. (D) The resulting force-indentation curves depend on holdfast height, showing convergence for thicker samples at small indentations (black, thickness of 120 nm; green, thickness of 60 nm; red, thickness of 12 nm). Download FIG S3, TIF file, 14.1 MB. Copyright © 2018 Hernando-Pérez et al. 2018 Hernando-Pérez et al. This content is distributed under the terms of the Creative Commons Attribution 4.0 International license.

At the surface molecular brush layer, the presence of regime I in the force-indentation curves is consistent with the presence of a very soft layer surrounding a much stiffer holdfast core (red in Fig. 2B, C, and D). The slow ramp data are absent in the curve obtained on a clean mica substrate, indicating that the phenomenon is indeed specific to tip-holdfast interactions. The first regime cannot be the result of electrostatics, which have a lower effective range (<60 nm).

We first hypothesized that the surface layer could be treated similarly to the holdfast core, with both regions described as elastic layers according to the Hertz model, albeit with different elastic moduli. However, creep compliance experiments that were performed at a low loading force (2 nN) and that corresponded almost exclusively to indentations of the surface layer revealed significant creep at constant load (Fig. 2E). Thus, unlike the stiff core, which is of a solid, elastic nature, the soft surface layer behaves as a viscoelastic material. As such, it is qualitatively different from the core material. While the validity of Hertz’s linear elastic theory does extend to viscoelastic materials when contact area is only weakly dependent on the loading rate (64), we have no evidence that this condition holds in our case, as the tip radius was generally smaller than the soft layer thickness. Moreover, attempts at using the Hertz model to describe both the surface layer and the bulk of the holdfast as elastic materials with different elastic moduli did not yield satisfactory fits to the AFM data over its full range (see Text S1 in the supplemental material).

Repulsive interactions similar to those obtained for the holdfast outer layer in magnitude and range have been previously observed where biopolymers are present on the surface of different bacteria (14, 65). In those cases, as well as with the holdfast, the magnitude and range of AFM interactions with the surface polymeric layer were much larger than those predicted by the theory proposed by Derjaguin, Landau, Verwey, and Overbeek (DLVO) (14, 65–67). The repulsive entropic force between two surfaces coated with polymer chains was derived by Alexander (68) and de Gennes (69) and adapted by Butt et al. (70) to describe the force experienced by a bare AFM tip as it probes a polymer brush. The forces measured on the outer holdfast layer (regime I) are indeed comparable with those predicted by this brush layer model. Hence, we have adopted the brush layer model as a possible explanation for the origins of the first regime observed (regime I). We have favored this approach over a continuous model of a cross-linked polymer mesh exhibiting a nonlinear stiffness profile (71, 72) because a continuous model would imply the presence of a well-defined holdfast/liquid interface extending around 50 to 100 nm from the substrate, while our data (Fig. S1) and those reported by others (40, 44, 45) show that the measured holdfast height is 40 nm on average, which corresponds to the stiff core regime in indentation experiments. We note that our model of two regimes consisting of a stiff core and an outer polymer brush layer does not necessitate a discontinuity in the force-indentation curves, as a compression of the brush layer would result in transmission of some fraction of the applied force to the core layer, yielding a continuous response (14, 65, 66, 73, 74).

Figure 2C shows a representative data set, where the first and second regimes are separately fitted to brush layer and linear spring models, respectively. In Fig. 2D, we show simultaneous fits to the data in these regimes using the brush layer and simple Hertz models (see Text S1 in the supplemental material). Both fitting approaches exclude a transition region at the surface of the holdfast (approximately 10% to 20% of the brush layer thickness) where the force response is neither purely elastic nor purely entropic in nature (see Text S1 in the supplemental material), and consequently the Hertz and brush layer models do not strictly apply. Lacking a model for the transition between the two regimes, it is not possible to obtain a satisfactory description of the data across the full range of cantilever deflection values without excluding this region (see Fig. S4 and Text S1 in the supplemental material). Using both fitting methods, as shown in Fig. 2C and D, similar estimates were obtained for the equilibrium length (*L*_0_) and density (Γ) of the brush layer (Table 1), and the data corresponding to the effective spring constant and Young’s modulus are consistent (see Text S1 in the supplemental material). Taken together, our results suggest that the holdfast consists of a stiff core with a Young’s modulus of 0.37 ± 0.18 GPa, decorated with a biopolymer brush with thickness of 89.8 ± 5.2 nm and ~10^17^ strands/m^2^ surface density (Table 1; see also red box and whisker plots in Fig. S5A to C). If we define the holdfast core as a hemisphere 40 nm in height (Fig. S1), our results imply the presence of fewer than ~1,000 exopolymeric strands on the surface of a holdfast. This is a very sparse, extended brush, which explains why it would be difficult to observe directly, by AFM or transmission electron microscopy (TEM). Note, however, the large force fluctuations that took place as compression occurred throughout the brush regime (Fig. 2B): the presence of sparse filamentous polymer molecules randomly interacting with the tip could provide a reasonable explanation for the observation of such significant fluctuations.

10.1128/mBio.02359-17.5 FIG S4  Hertzian and brush layer ﬁts to a representative 16-h data set. (A) Data determined by excluding the transition region at the surface of the holdfast, with ﬁt parameters given by *d*_0_ = 1.15 × 10^−9^ m, *Z*_0_ = 4.364 × 10^−6^ m, *E* = 1.79 × 10^9^ Pa, *L*_0_ = 2.15 × 10^−7^ m, and Γ = 2.37 × 10^16^ m^−2^. (B) Data determined by requiring the ﬁts to be continuous at the surface of the holdfast, with ﬁt parameters given by *d*_0_ = 5.08 × 10^−9^ m, *Z*_0_ = 4.367 × 10^−6^ m, *E* = 1.09 × 10^9^ Pa, *L*_0_ = 2.24 × 10^−8^ m, and Γ = 1.96 × 10^17^ m^−2^. The axis units are in meters. Download FIG S4, TIF file, 14.1 MB. Copyright © 2018 Hernando-Pérez et al. 2018 Hernando-Pérez et al. This content is distributed under the terms of the Creative Commons Attribution 4.0 International license.

10.1128/mBio.02359-17.6 FIG S5  Holdfast maturation characterization. Box and whisker plots of equilibrium length of polymer brush layer (A, D, and G), density of molecules on core surfaces (B, E, and H), core stiffness (C), and Young’s modulus (F and I) are shown for holdfasts incubated for 16 h (red) or 64 h (black). Data in panels A, B, and C were obtained from separated fits for the first and second regimes; data in panels D, E, and F were obtained from the fits by excluding the transition regimen between the holdfast bulk and the brush layer; and data in panels G, H, and I include the transition region. *n* = 310 and 298 for 16-h and 64-h holdfasts, respectively. *, *P* values < 0.025; ***, *P* values < 0.001 (by Mann-Whitney unpaired *t* tests). Download FIG S5, TIF file, 14.1 MB. Copyright © 2018 Hernando-Pérez et al. 2018 Hernando-Pérez et al. This content is distributed under the terms of the Creative Commons Attribution 4.0 International license.

### Modifications of holdfast mechanochemical properties. (i) Holdfast maturation.

Previous studies suggested that holdfast cures over time, strengthening adhesion with the substrate (38, 44, 45). To further assess the putative effect of aging on the holdfast structure, we calculated the core stiffness, equilibrium length (*L*_0_), and density (Γ) of the biopolymer brush layer after 16-h and 64-h incubation periods, from independent fits to the data in each of the brush layer and core regions, excluding the crossover regime (using the brush layer model fit for the first regime and extracting the slope of the linear dependence of the second regime, as described above). The biopolymer equilibrium length and density at 64 h show broader distributions with median values that are statistically different from those determined for the 16-h incubation (Table 1; see also Fig. S2B and black box and whisker plots in Fig. S5). The results are consistent with a long-term rearrangement of the brush layer, leading to the presence of a more compact biopolymer layer surrounding the core (65). In addition, the stiffness distributions were narrower at 64 h than at 16 h (Fig. S2 and S5), suggesting that the holdfast core becomes more homogeneous over time. Future experiments such as spatially resolved force curve analyses will allow a better characterization of the heterogeneous character of the holdfast and of its evolution over time.

### (ii) Influence of ionic strength.

*C. crescentus* is an oligotrophic bacterium usually found in habitats with low concentrations of solutes (34–36), suggesting that holdfast mechanochemistry is optimized for such solutions. Modifying the ionic strength of a solution can greatly impact the conformation of bacterial polysaccharides (75). We previously showed that increasing the ionic strength has a strong negative impact on bulk adhesion of the holdfast to surfaces (45), and here we investigated the influence of ionic strength on holdfast architecture (Fig. S6) (Table 1). Adding 10 mM NaCl modified the structure of the brush layer: its apparent length decreased by half, while the density of the polymeric strands doubled, suggesting a compaction of the overall brush layer (65). In addition, the holdfast core became twice as stiff in the presence of NaCl. These results are consistent with the 50% decrease in bulk adhesion of holdfast to glass in the presence of 10 mM NaCl (45) and argue that the holdfast adhesive is optimized for attachment in very-low-ionic-strength environments such as those from which *Caulobacter* bacteria are typically isolated (36).

10.1128/mBio.02359-17.7 FIG S6  Holdfast characterization in the presence of 10 mM NaCl. Box and whisker plots of (A) equilibrium length of polymer brush layer, (B) density of molecules on core surfaces, and (C) core stiffness for holdfasts incubated for 16 h in deionized (DI) water (red) or in DI water plus 10 mM NaCl (blue). *n* = 310 and 64 for DI water and DI water + NaCl-treated holdfasts, respectively. ****, *P* values < 0.00001 (by Mann-Whitney unpaired *t* tests). Download FIG S6, TIF file, 14.1 MB. Copyright © 2018 Hernando-Pérez et al. 2018 Hernando-Pérez et al. This content is distributed under the terms of the Creative Commons Attribution 4.0 International license.

### Role of the different holdfast components in structure and adhesion. (i) Core and brush layer properties.

In order to determine the chemical factors responsible for the observed mechanical properties, we performed nanoindentation measurements on holdfasts treated with three different enzymes: proteinase K (broad-range peptidase) to digest proteins and/or peptides; DNase I to digest both single-stranded and DNA double-stranded DNA (dsDNA) molecules; and lysozyme to digest 1,4 β-linked NAG residues (Fig. S7A). Figure S7 shows representative indentation curves corresponding to treated holdfasts (for comparison to the control nontreated holdfast representative curve shown in Fig. 2C).

10.1128/mBio.02359-17.8 FIG S7  Examples of fits for the indentation experiments on holdfasts treated with different enzymes. (A) Representative indentation curves of nontreated holdfasts (red) and holdfasts treated with proteinase K (green), DNase I (blue), and lysozyme (purple). (B) Zooms of the portions of the curves used to fit regime II. (C) Zooms of the portions of the curves used to fit regime I. (D to F) Representative indentation curves of holdfasts treated with proteinase K (D), lysozyme (E), and DNase I (F). The fits used to calculate the properties of the brush layer (regime I) and to calculate the stiffness of the holdfast core (regime II) are shown in red and blue, respectively. Download FIG S7, TIF file, 14.1 MB. Copyright © 2018 Hernando-Pérez et al. 2018 Hernando-Pérez et al. This content is distributed under the terms of the Creative Commons Attribution 4.0 International license.

**FIG 4:**
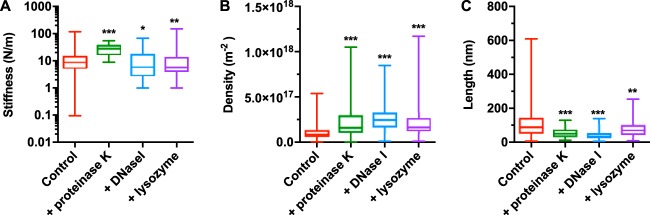
Indentation experiments on holdfasts treated with different enzymes show a change in core and brush layer properties. Box and whisker plots of (A) core stiffness, (B) polymer brush density, and (C) biopolymer brush equilibrium length. For all curves, examples of nontreated holdfast are represented in red and examples of holdfast after treatment with proteinase K, DNase I, and lysozyme are in green, blue, and purple, respectively. *n* = 310 for nontreated holdfasts and 87, 130, and 127 for holdfasts treated with proteinase K, DNase I, and lysozyme, respectively. *, *P* values < 0.025; **, *P* values < 0.01; ***, *P* values < 0.001 (Mann-Whitney unpaired *t* tests).

First, we studied enzymatic effects on the core stiffness, by fitting the linear part of the second regime of the indentation curves (Fig. S7B and D to F). The most marked differences with respect to the untreated holdfasts were observed after proteinase K treatment. The median stiffness value after proteinase K treatment was approximately three times higher than that determined for untreated holdfasts (Table 1). The stiffness distribution, however, was significantly narrower than that seen with the control and all other treatments (Fig. 4A). This result suggests that peptide residues present in the holdfast core are important for heterogeneity and elasticity. Holdfast stiffness dropped by a third under conditions of treatment with DNase I and lysozyme compared to that seen with nontreated holdfasts, suggesting that both DNA and NAG molecules may play a role in the constitutive properties of the holdfast core.

None of the enzymatic treatments entirely removed the long-range steric interactions between the tip and the holdfasts (Fig. S7C and D to F), but we observed quantitative changes in values for parameters Γ and *L*_0_ between treated and nontreated holdfasts (Fig. 4B and C). The most marked changes in brush parameters occurred after DNase I treatment. Equilibrium length *L*_0_ dropped to nearly a third of that of the control (Table 1). At the same time, the apparent density increased, suggesting a form of condensation (65). A similar trend was observed for proteinase K treatment, though to a lesser extent (45% decrease in length). Taken together, our results suggest that DNA and peptide residues are important components of the biopolymer brush layer. The impact of lysozyme treatment on the brush layer was less pronounced, with only a 30% decrease in biopolymer length, suggesting that the NAG residues might not be as important as the DNA or peptide entities with respect to the structure of the brush layer.

### Dynamic adhesion force spectroscopy.

**FIG 5:**
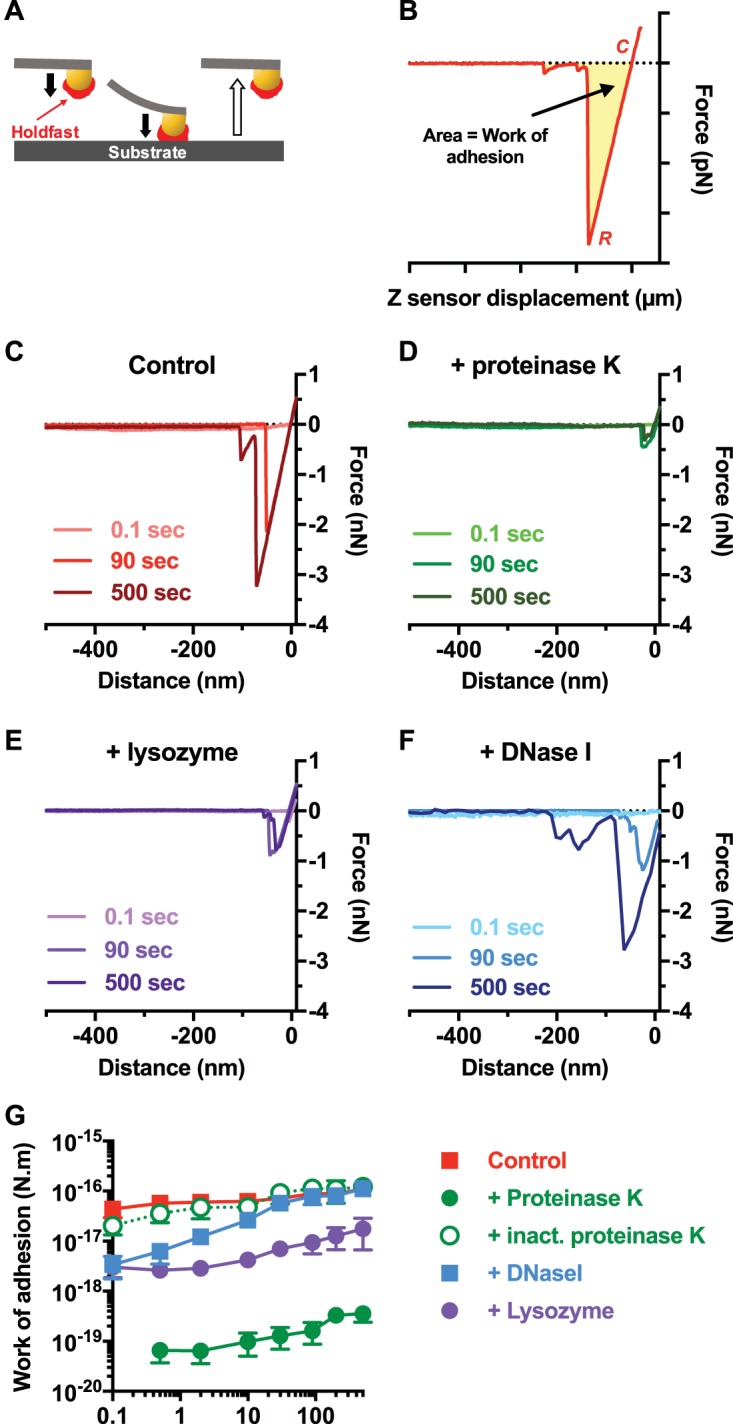
Dynamic adhesion force spectroscopy experiments performed with holdfasts treated with different enzymes show a change in holdfast adhesive properties. (A) Schematic of force spectroscopy experiments as described in the main text. (B) Representative force-displacement retraction curve collected on clean mica at 10 s of dwell time. The yellow area represents the work of adhesion. *C*, contact point; *R*, rupture point. (C to F) Representative curves obtained with a tip with immobilized nontreated control holdfast (C) and with holdfast treated with proteinase K (D), lysozyme (E), and DNase I (F). Curves recorded at 3 different dwell times (0.1, 90, and 500 s) are represented for each condition. (G) Work of adhesion of nontreated holdfasts (red) and of holdfasts treated with proteinase K (green), heat-inactivated proteinase K (green, open circles, dashed line), DNase I (blue), and lysozyme (purple) as a function of dwell time (fixed trigger point = 500 pN). Values are given as the averages of data from 10 samples determined in at least 4 independent experiments. The error bars represent standard errors of the means (SEM).

The indentation experiments performed on enzyme-treated holdfasts revealed the presence of peptide and DNA entities in the holdfast. To determine whether these constituents play a role in the strength of adhesion, we performed dynamic adhesion force spectroscopy experiments on a holdfast-coated tip and a clean surface as previously described (45). Holdfast-coated AFM tips were first treated with different enzymes, and the strength of adhesion between the treated tips and clean mica was measured at different dwell times (Fig. 5A). The adhesion forces were recorded as the work of adhesion, corresponding to the integrated area of the negative peak delimited by the contact point between the holdfast/AFM tip and the surface to the lowest deflection point of the curve (rupture force) (Fig. 5B). Representative curves, determined at different dwell times, corresponding to the control nontreated holdfast (Fig. 5C) and the holdfasts treated with the various enzymes (Fig. 5D to F) showed drastically different behaviors. The adhesion strength of enzyme-treated holdfasts was decreased under all conditions, to different degrees. Proteinase K treatment had a drastic effect on adhesion: a decrease of 3 orders of magnitude compared to the control sample was observed (Fig. 5D and G). This behavior was dependent on proteinase K enzymatic activity, since holdfast-coated tips incubated with heat-inactivated proteinase K exhibited the same adhesion as the nontreated control (Fig. 5G). This result suggests that peptide residues are important for initial holdfast adhesion strength. Exposure to lysozyme also led to a large (roughly 1 order of magnitude) decrease in adhesion, suggesting that NAG residues also participate in the adhesion of holdfast to surfaces (Fig. 5E and G). The results for DNase I-treated samples were more intriguing (Fig. 5F and G): while adhesion strength was diminished for short dwell times (rapidly occurring contact between the holdfast and the mica), DNase I-treated and nontreated tips behaved similarly at dwell times of higher than 10 s. This suggests that DNA molecules are involved in the initiation of adhesion but that their contribution to adhesion becomes comparably lower over time. Our results show for the first time that NAG residues are not the only components of the holdfast and highlight the presence of peptides and DNA molecules in the holdfast. These peptides and DNA molecules not only are part of the holdfast composition but also are crucial for its adhesiveness.

### Holdfast layer visualization.

High-resolution AFM images of holdfast in air confirmed the presence of long and thin structures associated with the holdfasts (Fig. S8A). However, due to the sparse (and possibly dynamic) nature of the brush, it is challenging to image the structures directly.

10.1128/mBio.02359-17.9 FIG S8  Holdfast visualization: (A) AFM topography images in air of two representative purified holdfasts adsorbed on mica. (B) Fluorescence microscopy of *Caulobacter* cells (top panels) and purified holdfasts (bottom panels) stained with TRSE (to label amine residues, red) and YOYO-1 (to visualize DNA, green). (C) Fluorescence microscopy of *C. crescentus* cells stained with AF647 WGA (to label NAG residues, red) and YOYO-1 (to label DNA, green). (Left panels) Nontreated cells. (Right panels) Cells incubated with 20 µg/ml DNase I for 1 h prior labeling and imaging. Plots on the right side represent the fluorescent pixel intensity plots for both the green and red channels. Download FIG S8, TIF file, 14.1 MB. Copyright © 2018 Hernando-Pérez et al. 2018 Hernando-Pérez et al. This content is distributed under the terms of the Creative Commons Attribution 4.0 International license.

We used fluorescence microscopy to visualize the different components in the holdfast on whole cells and in purified holdfasts (Fig. S8B). Texas Red succinimidyl ester (TRSE), which is an amine-reactive dye, labeled holdfasts attached to cells (Fig. S8B), but staining of purified holdfasts with TRSE was not successful (Fig. S8B), suggesting that this dye labels the holdfast anchor proteins present in the holdfast of wild-type cells but absent in shed holdfasts (76). The same result was obtained using fluorescent cysteine-reactive maleimide dye, and we have yet to identify a method to label the peptide components in shed holdfasts revealed by protease treatment and AFM experiments. However, we successfully stained holdfasts, including both the holdfasts on whole cells and purified holdfasts, using the DNA dye YOYO-1, confirming for the first time that DNA is a component of the holdfast (Fig. S8B). When we used wheat germ agglutinin lectin (WGA) to label the NAG residues (42) at the same time as YOYO-1, we observed colocalization of the two labels (Fig. S8C). The fluorescence intensity profile plots strongly suggest that the DNA molecules were in the outermost layer of the holdfast whereas the NAG residues were present in the inner layer (core). It is interesting that, when cells clustered in rosettes (i.e., interacted via their holdfasts), the DNA layer seemed larger than that seen on isolated cells. When cells were treated with DNase I prior YOYO-1 labeling and imaging, only faint labeling could be detected (Fig. S8C); the majority of the DNA molecules were easily accessible to the DNase I, suggesting that they were exposed and were hence in the brush layer.

**FIG 6:**
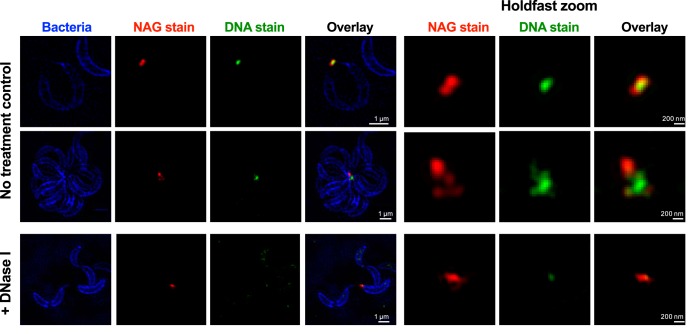
Structured illumination microscopy images of *C. crescentus* cells attached by their holdfast. Cells were stained using HADA (to label peptidoglycan), while NAG and DNA residues in the holdfast were labeled using AF-594 WGA (red) and YOYO-1 (green), respectively. Representative Z-stack images are presented. For the holdfast zoom panels, the average intensities for the entire stack for the green and red channels are projected together and merged in a single image.

By imaging labeled cells using superresolution, structured illumination microscopy (SIM) (Fig. 6), we were able to determine that NAG staining and DNA staining were clearly spatially segregated. In addition, it seems that holdfast-holdfast interactions were mediated by the DNA molecules. Indeed, we were able to detect several NAG patches in rosettes, probably corresponding to the cores of several holdfasts, interconnected by DNA molecules. Data revealing the origin of the DNA present in the holdfast, namely, revealing whether it is an intrinsic component of the secreted holdfast or represents extracellular DNA (eDNA) released in the culture when bacteria lyse, are still elusive. We previously showed that eDNA released during cell death can interact with holdfast and prevent adhesion (77), suggesting the intriguing possibility that the brush layer DNA is an intrinsic part of the holdfast that can be bound by eDNA released by cell death to inhibit adhesiveness.

### Conclusion.

The *Caulobacter* holdfast has impressive versatility in strongly binding wet surfaces of variable levels of roughness and hydrophobicity, which is hard to reconcile with the common understanding of holdfast as a simple NAG polymer. Here, using AFM dynamic force approaches, new structural and local chemical characteristics of the holdfast were unveiled which provide significant new insight into its properties.

Our data support a model where the holdfast is an organized as a two-layer system: a stiff core surrounded by a sparse and flexible polymer layer. Similar arrangements have been described in both prokaryotic and eukaryotic systems, such as bacteria covered with biopolymers (30, 74, 78) or human epithelial cells covered with cilia (61, 79). The holdfast stiff core, whose mechanical characterization suggests that it becomes more homogeneous over time, is surrounded by a sparse polymer layer that extends in the liquid over a distance that is several times the stiff core radius. The properties of the proposed outer brush layer are strongly affected by proteinase K and DNase I treatment, indicating that DNA and peptides are probable constituents of this brush layer. On the other hand, while peptides seem to be important for holdfast adhesion strength at all stages, DNA appears to be involved only in the initial adhesion step. However, we could not completely annihilate the holdfast adhesion strength or completely remove the brush layer. Measurement of indentation changes using holdfasts digested for various periods of time, different enzyme concentrations, or a combination of enzymes should help to further parse holdfast structure.

The layered holdfast structure reported here suggests a hypothetical model for holdfast adhesion wherein flexible brush layer fibers readily explore a surface, forming the first, initially weak contacts. Progressive compaction of the surface-bound brush layer could bring the holdfast core in increased contact with the surface. Stiffening of the core from its secreted fluid state to a hardness comparable to that of epoxy glues would then allow overall curing, which would help an applied force to be distributed among the surface bonds, thus reducing the load-dependent rate of detachment. A clear advantage of this complex structure and mechanochemistry is that each component can be modified to yield a diversity of adhesive strategies adapted to various environments. Other bacteria closely related to *C. crescentus* generate similar nanoscopic adhesive structures (18, 32, 33, 36, 42). Their habitats range from saltwater to freshwater to the rhizosphere, necessitating different conditions for effective adhesion. The studies and procedural framework described here should be useful in characterizing other bacterial bioadhesives, as well as in providing benchmarks for the development of future families of versatile, synthetic adhesives.

## MATERIALS AND METHODS

### *Caulobacter crescentus* purified holdfast sample preparation.

*C. crescentus* CB15 Δ*hfaB* (YB4251) (76) was grown in peptone-yeast extract (PYE) medium (36) at 30°C. In this strain, the gene encoding HfaB, a key protein for holdfast anchoring to the bacterial cell, is deleted, yielding a shedding phenotype: the holdfast is produced and exported outside the cell but fails to stay attached to the cell body (76). One hundred microliters of a *C. crescentus* Δ*hfaB* culture (optical density at 600 nm [OD_600_], 0.3 to 0.5) was spotted onto a freshly cleaved piece of mica and incubated at 30°C in a humid chamber. After a 16-h incubation in PYE medium, the mica surface containing shed holdfasts was carefully rinsed with sterile distilled water (dH_2_O) to achieve a low-ionic-strength condition typical of the oligotrophic environments from which *C. crescentus* is typically isolated. Indeed, the ionic strength of river and lakes where *Caulobacter* can be readily found attached to surfaces is very low and typically ranges between 1 and 5 mM (34–36). In addition, *Caulobacter* is readily found in potable and filtered water systems. These conditions are consistent with the conditions used in previous studies (45). This step removes the bacterial cells, while the shed holdfasts stay attached to the surface (45). One hundred microliters of sterile dH_2_O was placed on top of the holdfasts attached to the mica prior to AFM analysis. For 64-h-incubation-time samples, bacteria were removed from the surface after 16 h, as described above, and holdfasts attached to the mica were incubated in water in a humid chamber at room temperature for an additional 48 h. For holdfast enzymatic treatments, holdfasts were treated with the following enzymes: proteinase K, a nonspecific serine protease (10 µg/ml); lysozyme, a hydrolase specific for 1,4 β-links of NAG polymers (20 µg/ml); and DNase I, an endonuclease cleaving single-stranded and double-stranded DNA (10 µg/ml). Enzymatic treatments were performed for 1 h at room temperature and were applied to holdfasts bound to mica surfaces or immobilized to AFM tips.

### Atomic force microscopy experiments.

AFM measurements were performed using a commercial Cypher AFM instrument (Asylum Research) operating at room temperature.

### (i) Holdfast indentation and creep compliance experiments.

Samples were imaged using tapping-mode AFM. The AFM probe was driven near the first resonance frequency of its flexural mode and then engaged on the sample. The cantilever spring constant and quality factor of the first flexural mode were calibrated using the thermal noise method in liquid (80). The excitation frequency was chosen from the peak of the tuning curve, where the phase lag became 90°.

To corroborate the elasticity behavior of the holdfast core over time, we performed creep compliance experiments. In these experiments, the holdfast is deformed and the change of deformation over time, under constant load, is measured (50). First, a holdfast was centered in the scan area after imaging. Then, the AFM tip was moved onto the top of the particle and an *F*-versus-*Z* curve was acquired with a trigger force of around 40 nN to probe the holdfast core or of around 2 nN to probe the brush layer and a loading rate starting at 6.25 µm/s. During this first stage, the tip indented the particle until the selected trigger force was reached. Once the tip reached the set trigger force, the particle was deformed at a constant force using dwell times of 120 and 180 s, measuring the core and brush layer, respectively. Data from the *Z-*sensor channel were recorded to monitor the response of continuous deformation under conditions of a constant loading force. After each measurement, a new image was recorded to allow comparisons of the holdfast morphologies that were present before and after indentation. Images were processed using WSxM software (81).

For nanoindentation experiments in a liquid environment, a high-resolution image (360 nm by 360 nm, 128 points) of a given holdfast was recorded using amplitude modulation AFM (AM-AFM), in order to check the morphology and locate the center of the holdfast. We used a soft silicon nitride microcantilever (Olympus RC800) with a tip radius of 15 nm, a nominal spring constant of 0.3 to 0.4 N/m, and a resonance frequency of 69 kHz (~24 kHz in liquid) that was excited at an *A*_free_ amplitude of ~4 nm (*A*/*A*_ratio_ = 0.85 to 0.90). Once the holdfast was centered in the scan area, the AFM tip was moved to the top of the particle and a set of 3 to 8 force-displacement (*F* versus Z) curves (40 nN trigger force) was recorded at different locations within the same holdfast. For trigger forces of <5 nN, the holdfast exhibited linear elastic behavior. Force-versus-indentation curves (*F* versus Ind) were calculated according to the spring constants of the cantilever and according to the measurements of cantilever deflection on mica and on holdfast (82) (Fig. 3).

### (ii) Dynamic adhesion force spectroscopy experiments.

Dynamic adhesion force data were obtained from the interaction of a clean surface (freshly cleaved mica) and holdfast immobilized on the AFM tip in a liquid environment, as described previously (45), using silicon nitride gold-covered microcantilevers (MikroMasch HQ:CSC38) with a tip radius of <30 nm and a nominal spring constant of ~0.09 N/m.

First, we characterized the tip before each experimental set data. An *F*-versus-*Z* curve was determined using a clean mica surface with a clean AFM tip. The retraction curve showed characteristic adhesion between the tip and the mica. The holdfast was then immobilized on the same tip. To do so, a holdfast bound on the mica surface was first localized by imaging, and then some holdfast material was transferred to the AFM tip by the use of several contacts between the holdfast on the surface and the tip, using a dwell time of 90 s and a trigger force of 500 pN. Our choice of the trigger force was based our previous AFM study of holdfast (45) in which we tested adhesion forces of purified holdfasts using different trigger points spanning 1 order of magnitude on either side of this value (from 50 to 5,000 pN) and showed that the work of adhesion and maximal force measurements remained constant for trigger forces between 250 pN and 5 nN. To confirm the transfer, a second *F*-versus-Z curve was determined using clean mica. Only holdfast-covered tips which presented stronger adhesions than clean tips were further considered for our analysis. Once holdfast material was immobilized on the AFM tip, a new, freshly cleaved mica surface was used for determination of *F*-versus-*Z* curves, using different dwell times (0.1 to 500 s) and a trigger force of 500 pN. Data were analyzed as previously described (45).

### Nanoindentation data analysis.

For all AFM indentation experiments, 3 to 8 different measurements were taken at different locations in a single holdfast. For 16-h incubation samples, 86 holdfasts were measured in 10 independent experiments, yielding a collection of 310 *F*-versus-*Z* curves. For 64-h incubation samples, 59 holdfasts were measured in 8 independent replicates, yielding a collection of 298 *F*-versus-*Z* curves. For enzyme-treated holdfasts, 17, 34, and 25 different holdfasts were measured in at least 2 independent replicates for the proteinase K, DNase I, and lysozyme treatments, respectively, yielding collections of 87, 130, and 127 *F*-versus-*Z* curves, respectively. To characterize the interactions between the AFM tip and the holdfast, we converted the *F*-versus-*Z* curves into force-versus-distance tip-sample curves (*F* versus *D*; gap distance, *D* = *Z* − *Z*_0_ − *d*), where *Z* is the piezo displacement, *Z*_0_ is the point to contact, and *d* is the average cantilever normal deflection, as routinely performed in AFM spectroscopy for rigid surfaces (82, 83) (Fig. 3), using a script developed in-house on the basis of Igor Pro software.

We observed approximately linear behavior in the limit of large forces and characterized this approximately linear response of the holdfast bulk with an effective spring constant (*k*). We defined the contact point (*Z*_0_) between the tip and sample as the distance by which the *F*-versus-*D* curve departs from the linear behavior (14, 70, 82, 83). Holdfast deformation was negligible under conditions of forces lower than 5 nN (Fig. 3E). The strength and range of the tip-holdfast interaction forces measured in our experiments (forces close to 5 nN and a tip-sample interaction gap measured in a range of >50 nm) suggested that forces other than the classical van der Waals and electrostatic forces are involved, as described previously in other biological systems (14, 65). Indeed, those previous AFM measurements described the contribution of steric forces in various bacterial systems, where a biopolymer brush layer covering the bacterial cell surface is responsible for long-range interaction forces (14, 65, 84).

The AFM data determined over a low ramp range (2 to 60 nm) were fitted using the model previously developed to describe the interaction between an AFM tip and a grafted polymer surface (*F*_brush_) (70), based on theoretical work by Alexander (68) and de Gennes (69), as follows:

(1) $$F_{\text{brush}}\left(D\right)=50k_{B}TL_{0}{\text{Γ}}^{\frac{3}{2}}R_{\text{tip}}\exp\left(\frac{-2\pi D}{L_{0}}\right)$$

where *T* is temperature, *k*_*B*_ is the Boltzmann constant, *L*_0_ is the equilibrium length of polymer brush, *R*_tip_ is the tip radius (15 nm), Γ is the grafted polymer density, and *D* is the distance between tip and sample.

Using equation 1 and Igor Pro software, we estimated the equilibrium length *L*_0_ and the density Γ of the holdfast brush layer. Only curves with an acceptable fit (error < 10%) were considered for further analyses.

### Finite element calculations.

The finite element method was employed to simulate the indentation on films of different thicknesses on a stiff substrate. The three substrates were chosen to be 120 nm at the base. We imposed the homogeneous Dirichlet boundary condition at the bottom of the film. We applied a force at the top of the film and slowly (incrementally) increased the force at each iteration. We assumed the indenter to be a cylindrical end with a radius of 6.5 nm. The contact between the indenter and the film was assumed to be frictionless, and the body force was assumed to be zero. For all three cases, the Young’s modulus was set to be 0.2 GPa and the Poisson ratio was set to be 0.45. Moreover, a linear constitutive law was assumed to be appropriate.

### Simultaneous fits to Hertz and brush layer models.

For a spherical tip and for a small indentation, the Hertz model predicts the following relationship between force and indentation:

(2) $$F=\frac{4}{3}\frac{E}{1-\nu^{2}}{\text{δ}}^{3/2}\sqrt{R^{*}}$$

where *E* is the Young’s modulus of the holdfast; *ν* is the Poisson ratio (taken to be 0.5 here, assuming incompressibility of the holdfast), and R* = *R*_tip_ *R*_sample_/(*R*_tip_ + *R*_sample_) where for typical sample radii, R* = ≈*R*_tip_. The sample indentation (δ) is given as follows:

(3) $$\text{δ}=Z-Z_{\text{0}}-(d-d_{0})$$

where *d* is the cantilever deflection, *Z* is the piezo height, *d*_0_ is the deflection of the cantilever far from the sample, and *Z*_0_ represents the piezo displacement for which the cantilever touches the surface of the holdfast core. The force was obtained from the cantilever deflection using the cantilever spring constant as *k_c_*: *F* = *k_c_* (*d* − *d*_0_).

We extracted parameters describing holdfast material properties from the raw AFM data by performing simultaneous least-squares fits to Hertz and brush layer models (Table 1). Specifically, with the cantilever deflection given by *d* and the piezo height given by *Z*, we plotted *Z* − *d* versus *d* and fitted the data in selected regions to functional forms describing the bulk of the holdfast and a surface brush layer, as described below. We chose to plot the data this way because the fit procedure is most robust for the portion of the data at large *d* values, where *Z* − *d* varies slowly with *d*, allowing reliable extraction of the modulus of elasticity (*E*).

We followed two approaches to fit the AFM data simultaneously to the Hertz and brush layer models. In the first approach, we fitted the brush layer model all the way to the surface (see Text S1 in the supplemental material), where we allowed the boundary between these regions to float as a fit parameter in the least-squares minimization process. For values of *d* greater than this value, the fit function is the Hertzian form, given as

(4) $$Z-d={\left(\frac{d-d_{0}}{\text{γ}}\right)}^{\frac{2}{3}}+Z_{0}-d_{0}$$

where the Young’s modulus is obtained from the fit parameter Γ as

(5) $$E=\frac{3\text{γ}}{4}\frac{k_{c}}{\sqrt{R_{\text{tip}}}}\;\left(1-\nu^{2}\right)$$

For values of *d* lower than the boundary value, the fit function represents a description of the brush layer as

(6) $$Z-d=\frac{L_{0}}{2\text{π}}\text{ln}\left(\frac{d-d_{0}}{\text{α}L_{0}}\right)+Z_{0}-d_{0}$$

where the brush layer density is obtained from the fit parameter α as

(7) $$\text{Γ}={\left(\text{α}k_{c}/50k_{b}TR_{\text{tip}}\right)}^{2/3}$$

In the second approach, we excluded the transition region, corresponding to *D*/*L*_0_ values of less than ~0.1 to 0.2, from the brush layer fitting region, where the force is not strictly entropic. In the supplemental material, we show that the two approaches yield results for the values of fitted parameters that are consistent within an order of magnitude (Table 1). However, we emphasize that the brush layer is highly compressed in the transition region between the bulk of the holdfast and the surface layer and that neither model produces results that constitute an accurate physical description.

### Fluorescence microscopy.

Holdfasts were fluorescently labeled and visualized on whole *C. crescentus* CB15 wild-type cells. Exponential cultures (OD_600_ = 0.4 to 0.7) grown in PYE medium (36) were stained using 0.5 µg/ml Alexa Fluor 647-conjugated wheat germ agglutinin lectin (AF647-WGA; Molecular Probes) and 1 µM YOYO-1 DNA stain (Molecular Probes) and subjected to 5 min of incubation at room temperature. WGA specifically binds to the NAG residues present in the holdfast (42), while YOYO-1 is a cell-impermeant dye that has a high affinity for dsDNA molecules. For staining using Texas Red succinimidyl ester (TRSE; Molecular Probes) (amine-reactive dye), cells were mixed with 5 µg/ml dye (1/1,000 dilution in 100 mM NaCO_3_ buffer, pH 8) and incubated for 20 min at room temperature before being washed 3 times by centrifugation (3,000 × *g* for 2 min) and resuspended in dH_2_O. One microliter of labeled cells was spotted onto a 24-mm-by-60-mm microscope glass coverslip and covered by an agarose pad (1% in water). Samples were imaged by epifluorescence microscopy using an inverted Nikon Ti-E microscope with a Plan Apo 60× objective, an Andor iXon3 DU885 EM charged-coupled-device (CCD) camera, and Nikon NIS Elements imaging software.

### Transmission electron microscopy (TEM).

Exponentially grown *C. crescentus* CB15 wild-type cells were spotted onto Formvar-coated, carbon film-stabilized copper grids (Electron Microscopy Sciences) and incubated for 1 h. Each grid was washed with dH_2_O, negatively stained with 7.5% uranyl magnesium acetate for 5 min, and washed five times with dH_2_O again. Imaging was performed with a JEOL JEM-1010 transmission electron microscope set to 80 kV.

### Structured illumination microscopy (SIM).

For SIM imaging, we used HADA, a blue fluorescent d-amino acid (85), to label *C. crescentus* CB15 peptidoglycan; YOYO-1 (Molecular Probes) to label dsDNA present in the holdfasts; and AF594-WGA (Molecular Probes) to label NAG residues present in the holdfast. One milliliter of *C. crescentus* CB15 was grown to exponential phase in PYE medium and labeled with 1 µM YOYO-1 and 0.5 µg/ml AF594-WGA for 5 min at room temperature. Cells were washed 5 times by centrifugation (3,000 × *g* for 2 min) in 1 ml PYE medium to remove all traces of unbound YOYO-1 and AF594-WGA. HADA (1 mM) was added to the washed cells in 1 ml PYE medium. After 25 min of incubation at 30°C under conditions of constant shaking (200 rpm), cells were washed again 3 times by centrifugation (3,000 × *g* for 2 min) to remove unbound HADA and resuspended in 50 µl of water. One microliter was spotted onto a 24-mm-by-50-mm microscope glass coverslip and covered by an agarose pad (1% in water) before imaging was performed. Because of the multiple wash steps performed during the labeling process, all cells harboring a holdfast were arranged in rosettes, i.e., clusters of cells interacting via their holdfasts, and we were unable to detect isolated cells with a holdfast.

Z-series images of cells harboring a holdfast images were acquired on a DeltaVision OMX three-dimensional (3D) SIM superresolution system (Applied Precision Inc.) equipped with an inverted 1.4 NA Olympus 100× oil objective. Images were processed (deconvolution and alignment) using Softworx imaging software.
