# Supplementary figures and images for: Layered Structure and Complex Mechanochemistry Underlie Strength and Versatility in a Bacterial Adhesive
Source: mBio. 2018 Feb 6;9(1):e02359-17. doi: 10.1128/mBio.02359-17 (PMC5801468; doi:10.1128/mBio.02359-17)

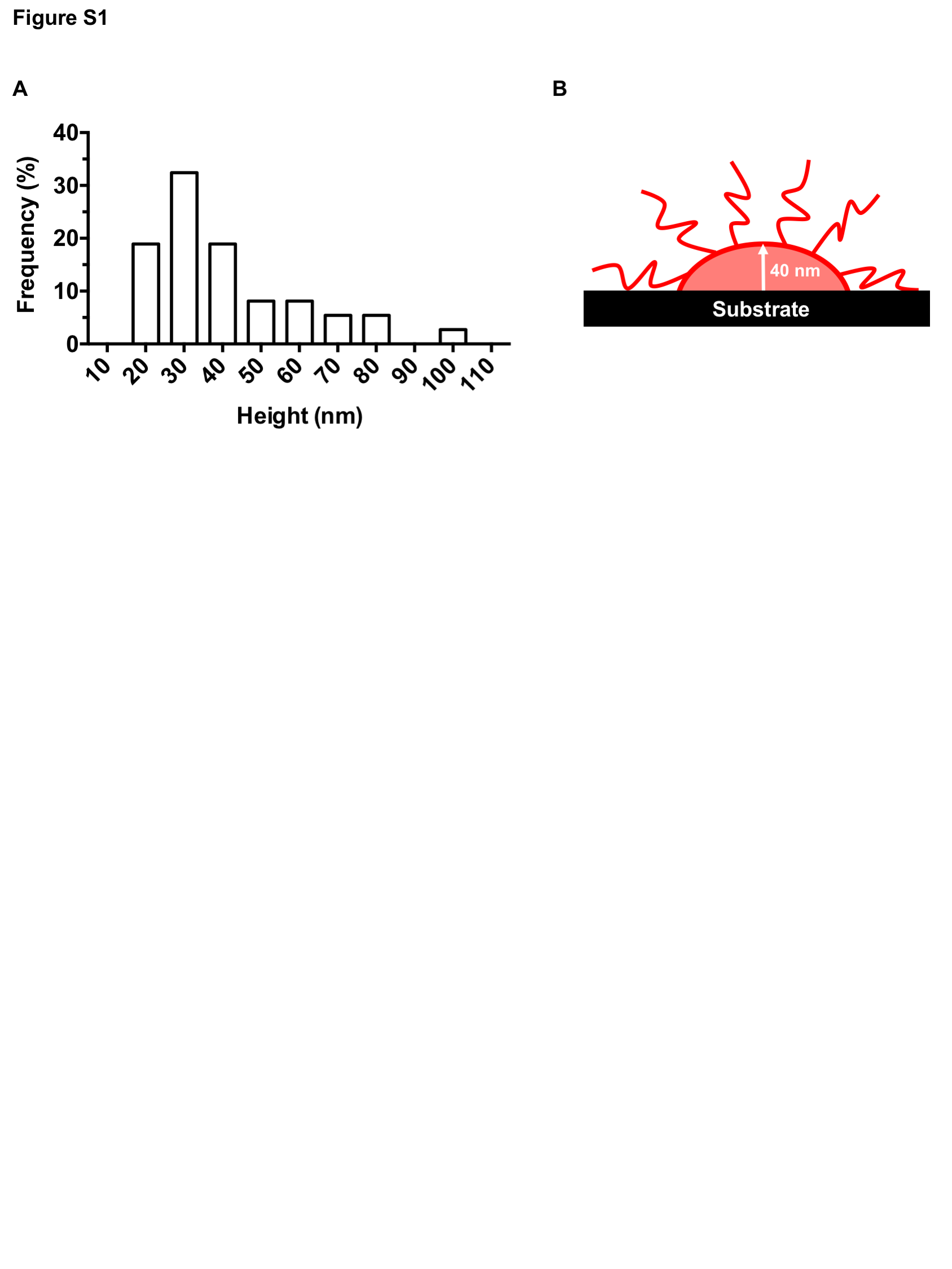

Supplement: FIG S1 [file mbo001183709sf1.tif]

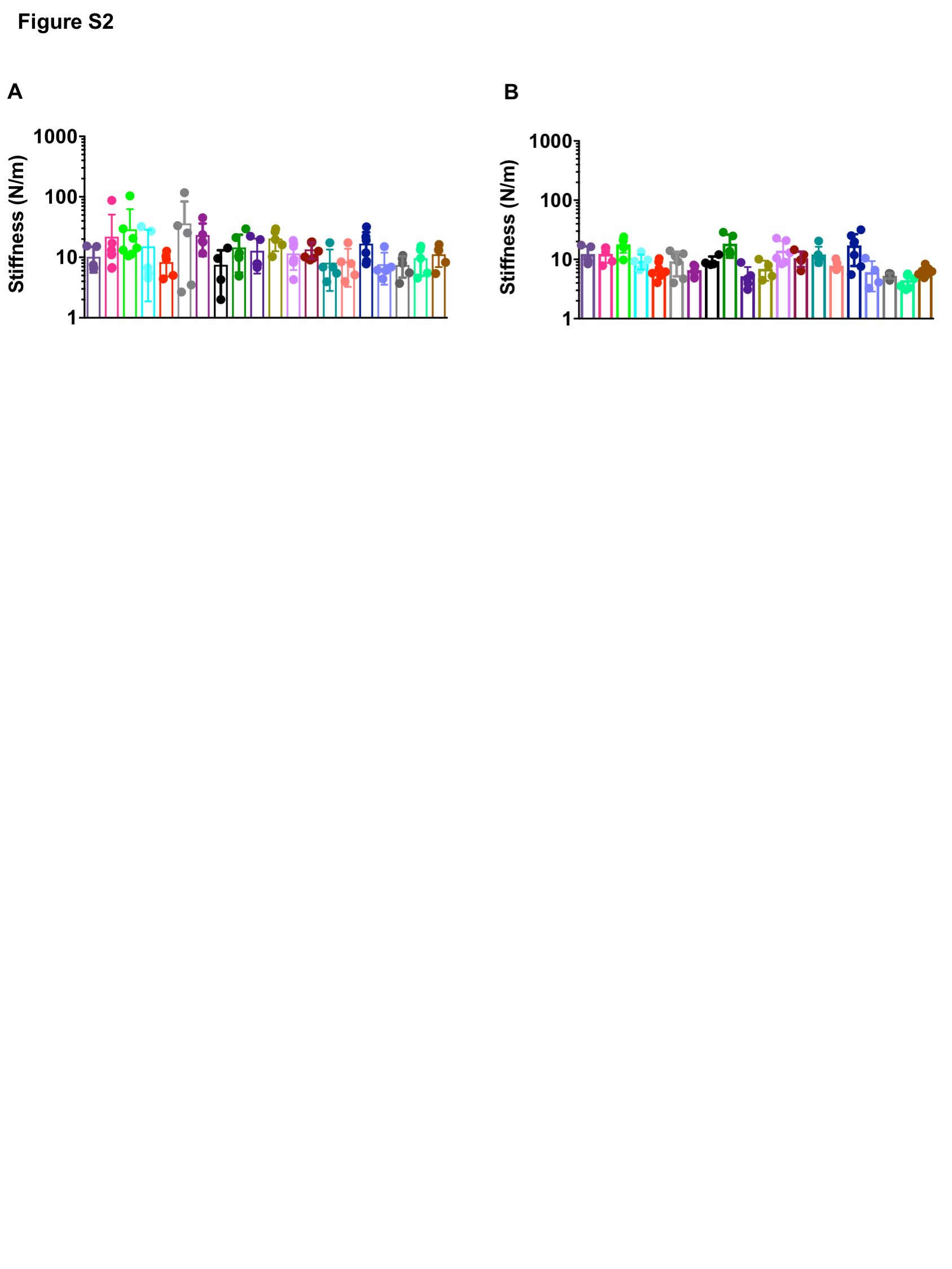

Supplement: FIG S2 [file mbo001183709sf2.tif]

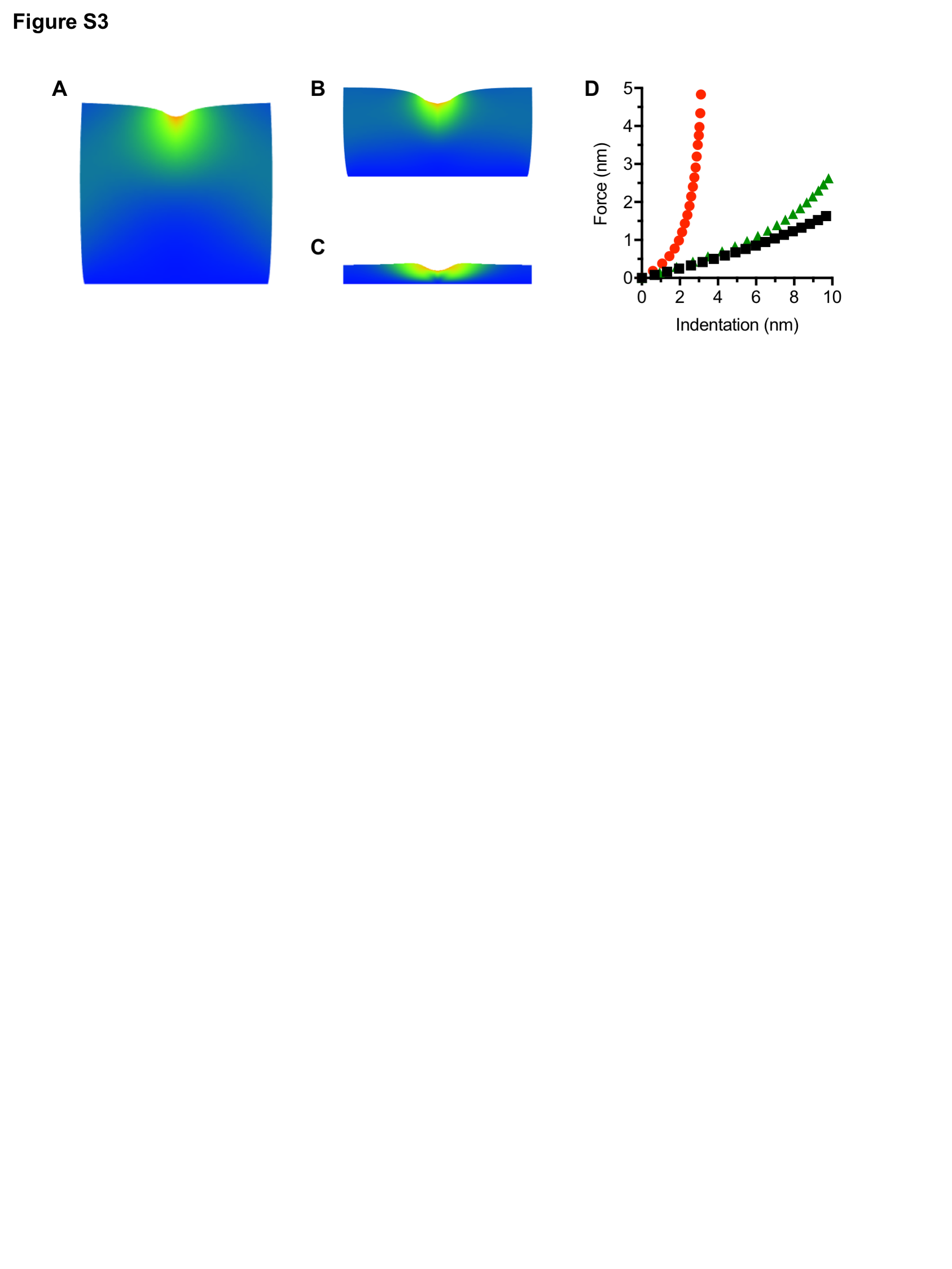

Supplement: FIG S3 [file mbo001183709sf3.tif]

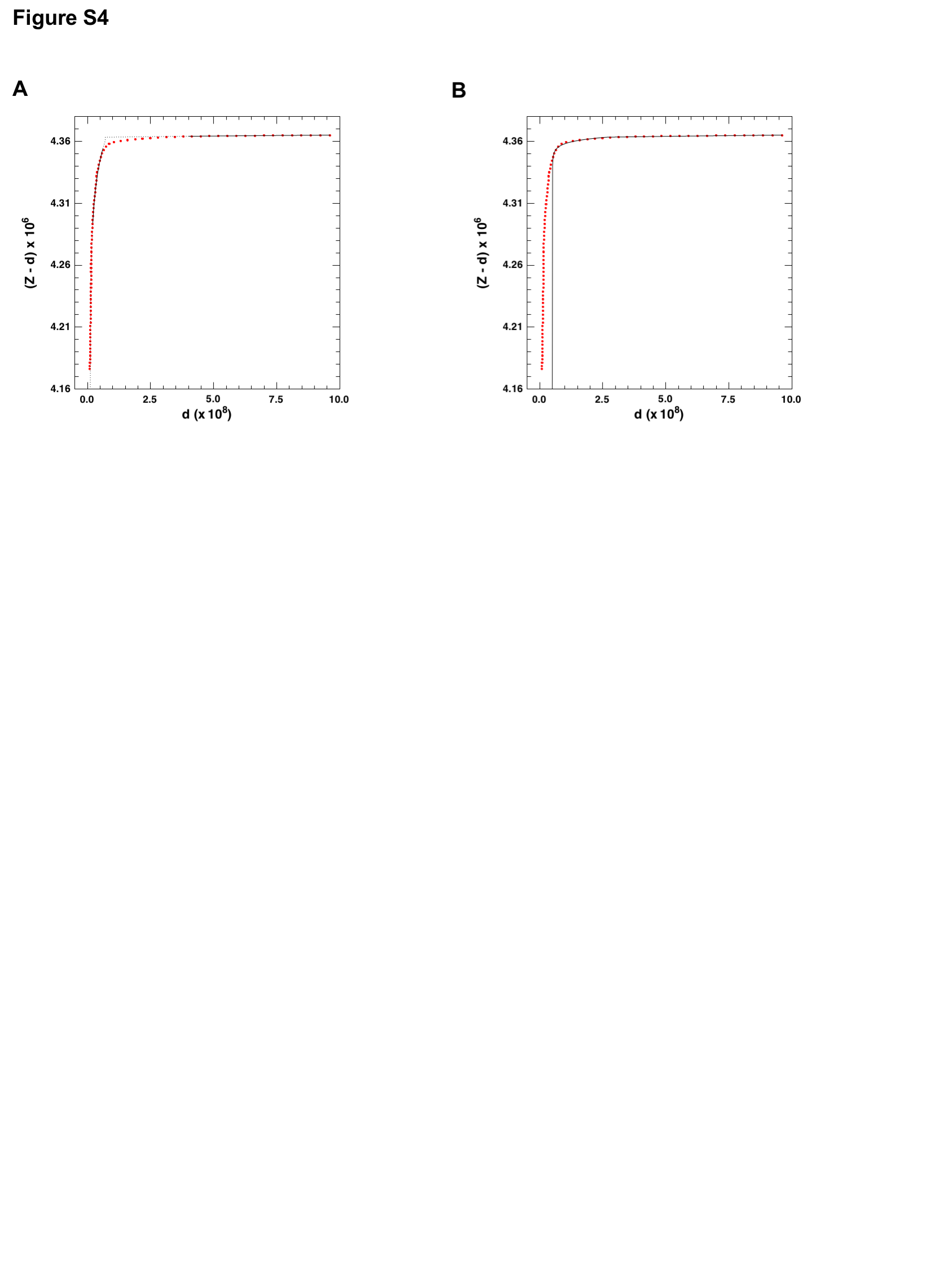

Supplement: FIG S4 [file mbo001183709sf4.tif]

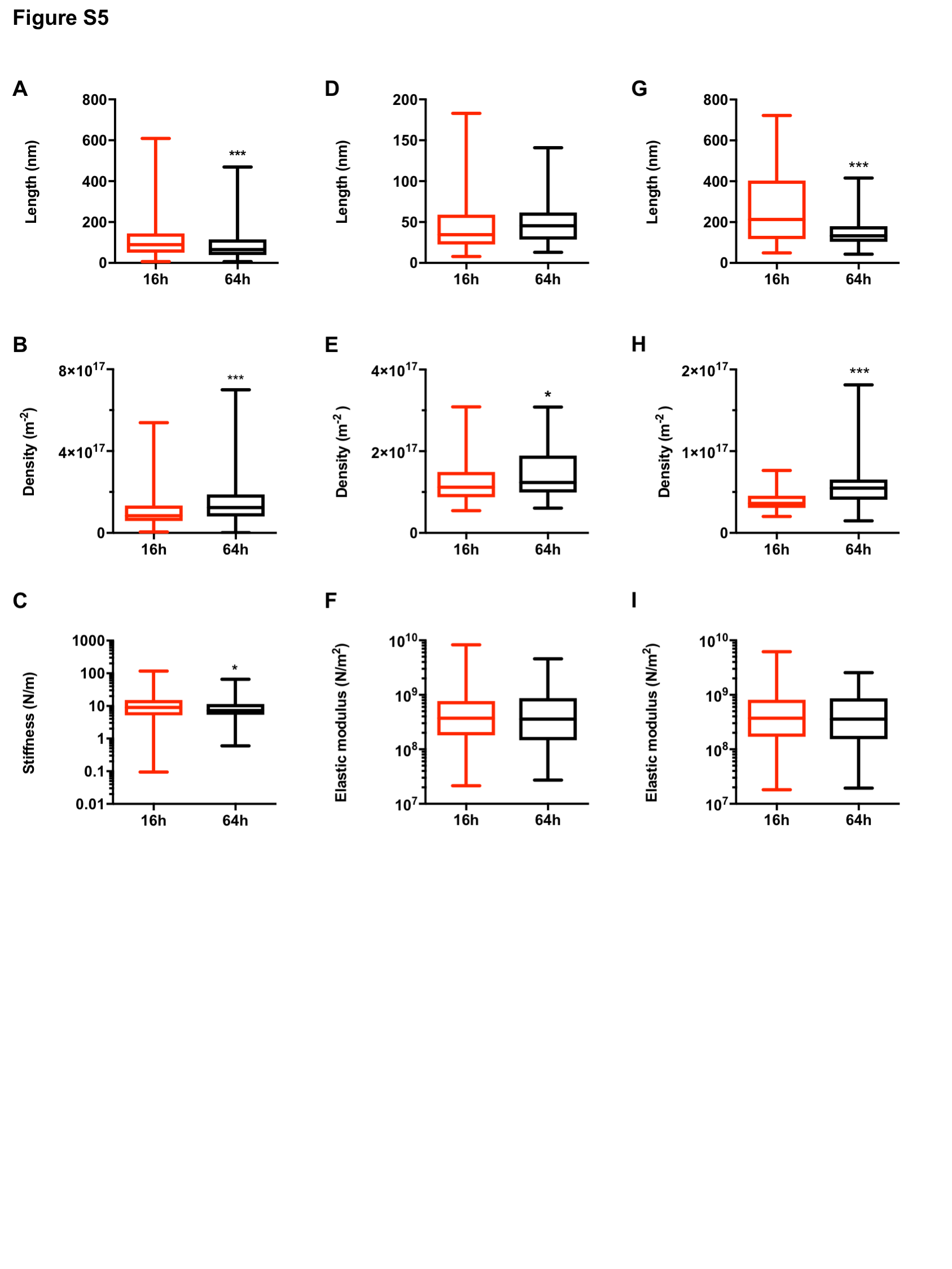

Supplement: FIG S5 [file mbo001183709sf5.tif]

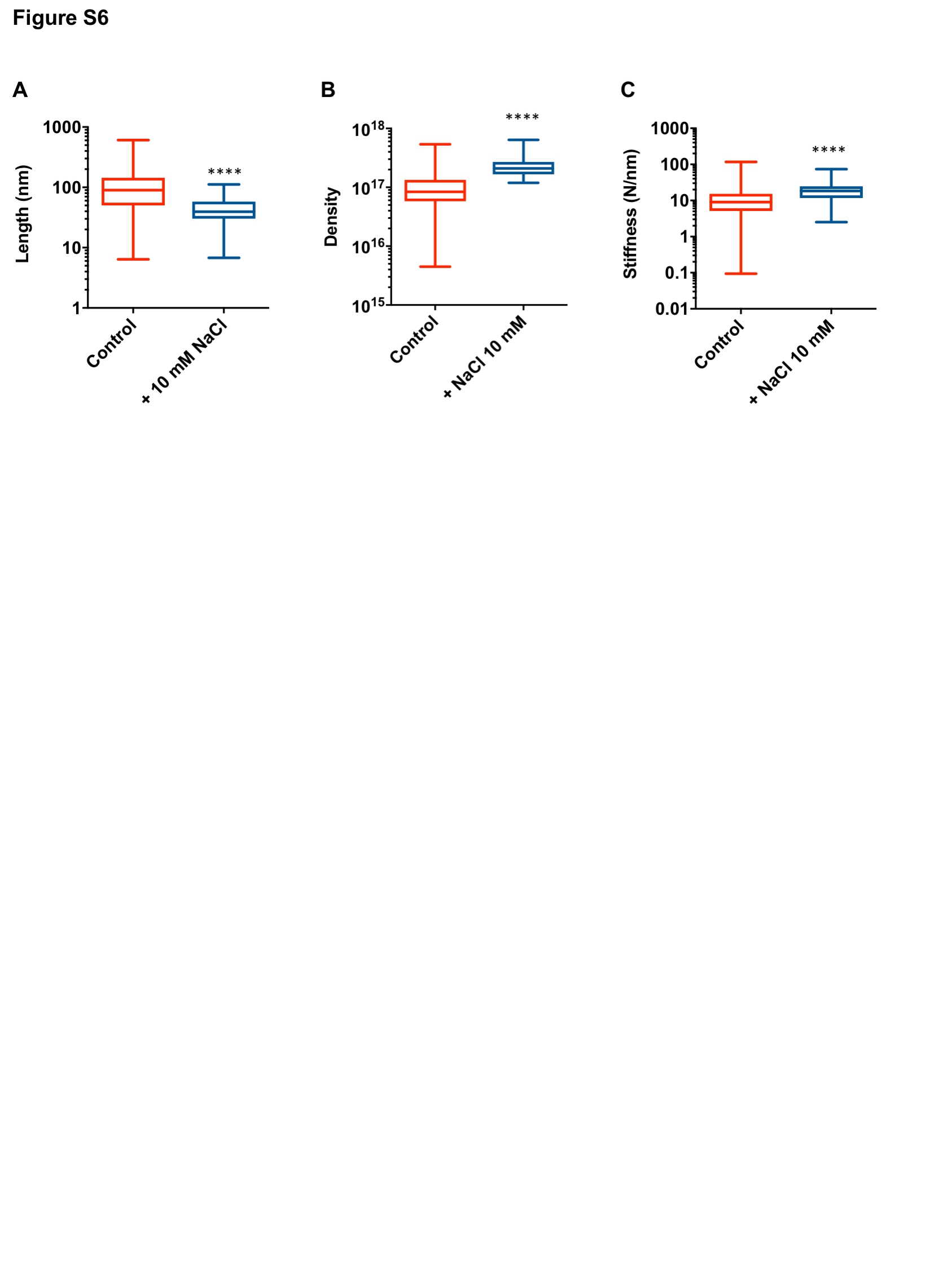

Supplement: FIG S6 [file mbo001183709sf6.tif]

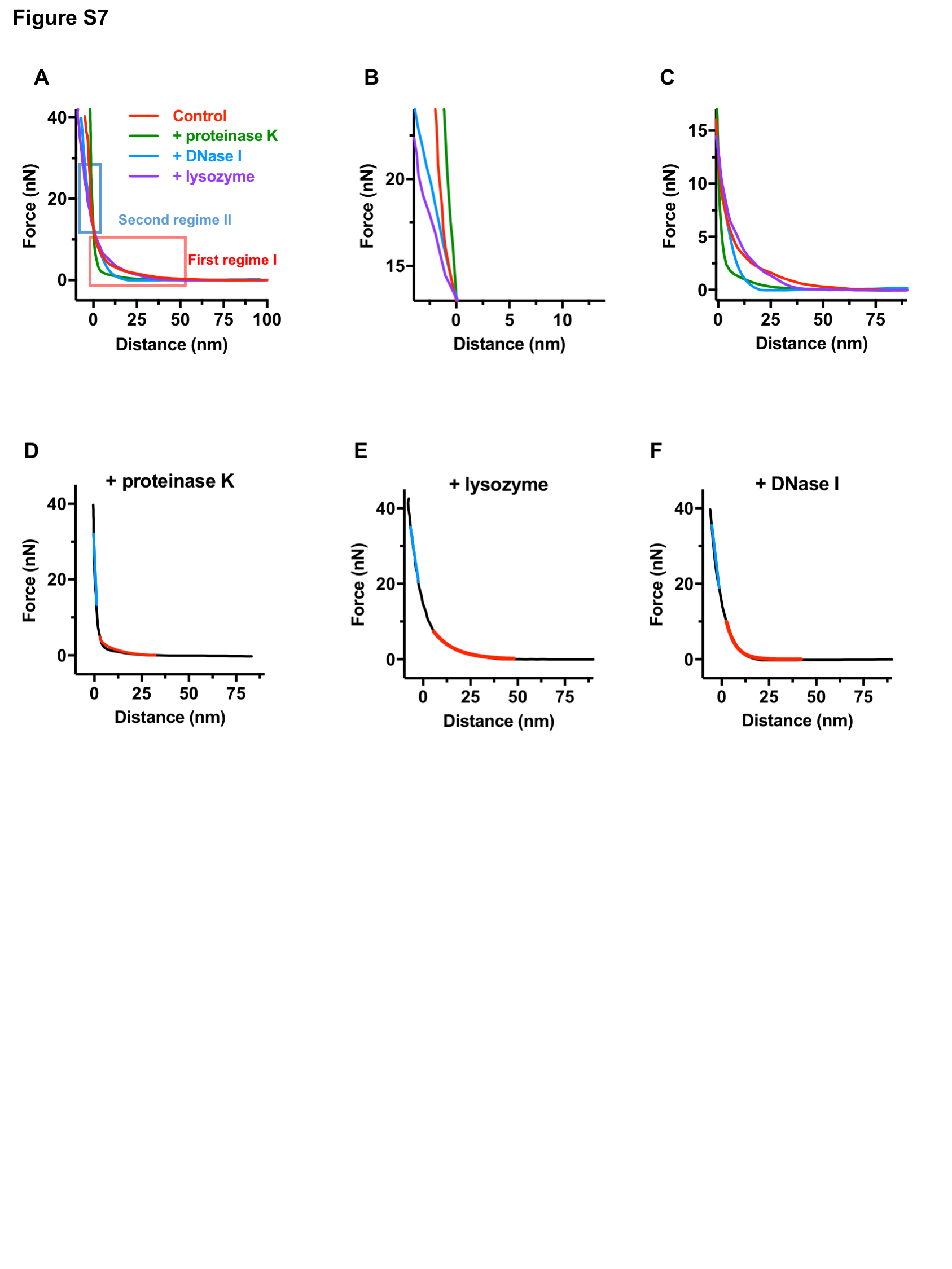

Supplement: FIG S7 [file mbo001183709sf7.tif]

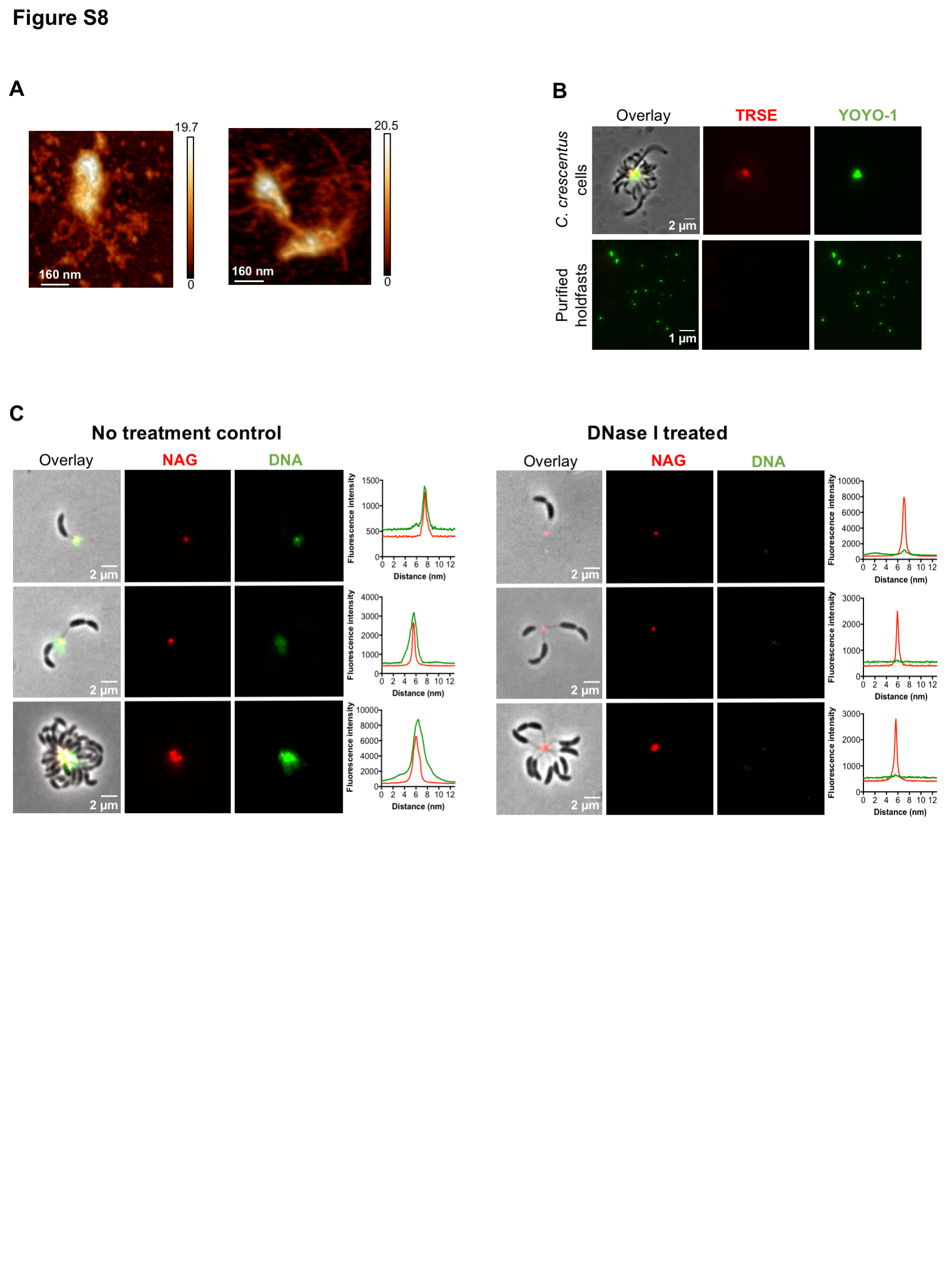

Supplement: FIG S8 [file mbo001183709sf8.tif]
