# Supplementary material for: Layered Structure and Complex Mechanochemistry Underlie Strength and Versatility in a Bacterial Adhesive
Source: mBio. 2018 Feb 6;9(1):e02359-17. doi: 10.1128/mBio.02359-17 (PMC5801468; doi:10.1128/mBio.02359-17)
Supplement: TEXT S1 [file mbo001183709s1.pdf]

Supplementary Material

Multi-regime description of holdfast material properties

The Hertz model describing the elastic response of soft materials is used to quantify the bulk of the holdfast. For a spherical tip and for small indentation of the sample [1], it is given by

$$F = \frac{4}{3}\frac{E}{1-\nu^2}\delta^{3/2}\sqrt{R^*}, \tag{1}$$

where E is the Young's modulus of the holdfast; ν is the Poisson ratio, taken to be 0.5 here, assuming incompressibility of the holdfast, consistent with previous works showing the holdfast to be a watery material [2]; $R^* = R_{tip}R_{sample}/(R_{tip} + R_{sample})$, where for typical sample radii, $R^* \approx R_{tip}$. The sample indentation, δ, is given by [3]

$$\delta = Z - Z_0 - (d - d_0), \tag{2}$$

where d is cantilever deflection, and Z is the piezo height; d_0 is the deflection of the cantilever far from the sample, and Z_0 represents the piezo displacement for which the cantilever touches the surface of the holdfast. The force is obtained from the cantilever deflection using the cantilever spring constant, k_c: $F = k_c(d - d_0)$. In our fits, d_0 and Z_0 were treated as fitting parameters.

This simple form of the Hertz model assumes the sample is homogeneous, isotropic and infinitely thick, whereas real biological samples deviate from these idealized conditions. Finite thickness corrections to the Hertz model have been proposed [5, 6], and in Section 3, we compare fit results with and without correction terms accounting for the finite thickness of the holdfast.

As justified below, the Hertz model alone does not describe the holdfast AFM data over its full range. Rather, in addition to the holdfast behaving as a material with a (hard) elastic bulk, the data suggest the existence of a surface force and associated surface layer [7]. Other works have also considered extensions of the Hertz model in characterizing material properties of cells, where the AFM tip probes a pericellular "brush" layer consisting of surface protrusions and a glycocalyx before physically encountering the cell surface itself [4, 8].

A surface coated with polymer chains, such as that of a cell or the holdfast, experiences a repulsive entropic force upon approach of a second surface, such as an AFM tip, due to the reduced conformational entropy of the chains or brushes. For two brush layers each with thickness L_0 closer than a distance $2L_0$ from each other, the repulsive pressure is given according to the Alexander-de Gennes theory [9, 10, 11] as

$$f(D) = k_B T \Gamma^{3/2}\left[(2L_0/D)^{9/4} - (D/2L_0)^{3/4}\right], \tag{3}$$

where D is the distance between the surfaces, Γ is the brush layer density, k_B is the Boltzmann constant, and T is temperature. It can be shown that in the range $0.2 < D/2L_0 < 0.9$, the expression in Eq. 3 is approximately exponential

$$f(D) \approx 100 k_B T \, \Gamma^{3/2} e^{-\pi D/L_0}. \tag{4}$$

For a bare spherical tip interacting with a polymer brush, the pressure is obtained by substituting $L_0/2$ for L_0, and dividing the pressure by 2. Using the Derjaguin approximation, the total force for a parabolic tip profile ($Z - D = r^2/2R_{tip}$) becomes [12]

$$F(D) \approx \int_0^\infty f \, 2\pi r dr = 2\pi \int_D^\infty f(Z) \, r \frac{dr}{dZ} dZ = 2\pi R_{tip} \int_D^\infty f(Z) dZ = 50 k_B T \Gamma^{3/2} L_0 R_{tip} e^{-2\pi D/L_0}. \tag{5}$$

The validity of brush models is usually evaluated retrospectively after finding the brush parameters, in particular L_0. When the AFM probe-surface distance, D, is less than approximately 20% of the brush layer thickness, L_0, the brush layer response is no longer strictly entropic in nature. Recent work has shown that at high compression, the brush behaves as an elastic layer [4], contributing to an effective elastic modulus for the underlying sample.

1 Simultaneous fits to Hertz and brush layer models

We extract parameters describing holdfast material properties from least-squares fits to the raw AFM data given by cantilever deflection, d, and piezo height, Z. Specifically we plot $Z - d$ versus d, and fit the data in selected regions to functional forms describing the bulk of the holdfast and a surface brush layer, as described below. We chose to plot the data in this way in order that the fit procedure is most robust for portion of the data at large d, where $Z - d$ varies slowly with d, allowing reliable extraction of the modulus of elasticity, E, which constitutes a central result of our work.

In this section, we describe two approaches to fitting the AFM data: In the first approach, we fit the brush layer model all the way to the surface. In the second approach, we exclude the transition region corresponding to D/L_0 less than $\sim 0.1 - 0.2$, wherein the brush layer force is not strictly entropic. Our results demonstrate that while the Young's modulus characterizing the material properties of the bulk of the holdfast is different in these two approaches, the results are consistent and vary by less than an order of magnitude. However, we emphasize that in the transition region at the surface of holdfast, neither the Hertz nor brush layer model strictly holds.

In using the Hertz and brush layer models to describe the stiff elastic bulk region coated with biopolymer, first we allow the boundary between these regions to float as a

free parameter in the least-squares minimization process. For values of d greater than this value, the fit function is the Hertzian form, given by

$$Z - d = \left(\frac{d - d_0}{\gamma} \right)^{2/3} + Z_0 - d_0 \,,$$

where the Young's modulus is obtained from the fit parameter γ as $E = \frac{3\gamma}{4} \frac{k_c}{\sqrt{R_{tip}}} \left(1 - \nu^2 \right)$. For values of d less than the boundary, the fit function represents a description of the brush layer,

$$Z - d = \frac{L_0}{2\pi} \ln \left(\frac{d - d_0}{\alpha L_0} \right) + Z_0 - d_0 \,,$$

where the brush layer density is obtained from the fit parameter α as $\Gamma = (\alpha k_c / 50 k_B T R_{tip})^{2/3}$.

To allow for an assessment of goodness of fit via a χ^2 metric, we assign an uncorrelated $0.25\, nm$ experimental uncertainty to every value of $Z - d$, based on empirical evaluation of instrumental drift over the measurement time frame. This value represents an upper bound, in the sense that both the point-by-point fluctuations and perceived unphysical drifts within measurements of most samples are smaller than this. For our fits we have not incorporated the corresponding uncertainties on what we take as the independent observable (d). We have excluded from the fits the points with $(d - d_0) \sim 0$, since they fall outside the range of applicability of the brush layer model ($D > 0.9 L_0$).

With the approach described above, it is possible for most samples to obtain fits to the data with satisfactory χ^2 values, in which the model used for the brush layer gives a prediction for $Z - d$ that matches that of the Hertzian model at the boundary between the two. However we have also observed that (1) the values of L_0 are driven to be progressively smaller than what we find when we successively include more data points at low $d - d_0$ values, and (2) in some cases the best-fit values for d_0 deviate significantly from what is expected based on visual inspection of the low $(d - d_0)$ region. This deviation is likely due to the fact that by requiring the fits based on the two functions to be continuous at the surface of the holdfast where $D < 0.2 L_0$, the brush layer is highly compressed and the model does not strictly hold, as described above.

As a result we have also carried out fits where, in addition to points at very low $d - d_0$, we exclude data points in the transition region approximately centered on the boundary between the brush layer and bulk behaviors. By excluding the transition region from the fit, the two problems noted above are avoided. Determination of the limits of the excluded region is done empirically on a sample-by-sample basis, but is not highly tuned in order to avoid the introduction of significant biases that might result from fine-tuning based on the data itself.

We show representative fits to 16 h and 64 h data where the transition region at the surface is excluded (Fig. A and Fig. C, respectively) and the brush layer and Hertz models

are continuous at the surface of the holdfast (Fig. B and Fig. D, respectively). In Table 1, we summarize fit results for the parameters characterizing the material properties of the holdfast (E, L_0, Γ) using the two fitting approaches.

2 Surface layer as an elastic material

We also investigated a description of the data wherein the surface layer and bulk of the holdfast are both characterized as elastic materials and described by the Hertzian model, with different elastic moduli. We find that the data does not support this description as well as the brush layer model for the surface layer. As an example, in Fig. E and Fig. F, we show typical fits to the same 16 h and 64 h data sets (and using the same data points) as in Fig. A and Fig. C, where the transition region at the surface has also been excluded. We note that the values of χ^2 per degree of freedom are larger for the Hertzian model of the surface layer relative to the brush layer model. If the transition region is included (and the Hertzian model of the surface layer is extended all the way to the surface of the holdfast, analogous to the brush layer model fits in Fig. B and Fig. D), we are not able to obtain satisfactory fits (results not shown).

3 Finite thickness correction to Hertz model

In Fig. G-H and Fig. I-J, we show fits to the bulk region, including higher order corrections to the Hertz model for a semi-infinite sample to account for the fact that for finite thickness samples, denoted by height h, the presence of the rigid substrate modifies the pressure distribution across the contact area between the probe and sample. Using the method of images to satisfy the boundary conditions at the rigid boundary, the general result has been obtained for the Green's function for the deformation of the finite sample free surface [5, 6], and for a spherical tip in the limit of small indentation ($\delta/h \ll 1$), it is given by:

$$F = \frac{4}{3}\, E\, R_{tip}^{1/2}\, \delta^{3/2} \left[1 - \frac{2\alpha_0}{\pi}\chi + \frac{4\alpha_0^2}{\pi^2}\chi^2 - \frac{8}{\pi^3}\left(\alpha_0^3 + \frac{4\pi^2}{15}\beta_0\right)\chi^3 + \frac{16\alpha_0}{\pi^4}\left(\alpha_0^3 + \frac{3\pi^2}{5}\beta_0\right)\chi^4\right], \tag{6}$$

where $\chi = \sqrt{R_{tip}\delta}/h$. Parameters α_0 and β_0 depend on the Poisson ratio, ν, and for an incompressible sample ($\nu = 0.5$) adhered to substrate, the values are $\alpha_0 = -1.7795$ and $\beta_0 = 1.0079$.

In Fig. G-H, we show fits for representative data sets which constitute samples with approximately equal elastic moduli but varying sample heights (corresponding to the thin and thick ends of the holdfast height distribution). We note that consistent with the trend

in the finite element simulations of holdfast deformation (Figure S3), the change in holdfast elastic modulus accounting for its finite thickness is smaller (larger) for thicker (thinner) holdfasts. Similarly, in Fig. I-J, for representative samples with approximately equal heights but varying elastic moduli, we find that with increasing sample stiffness, this ratio decreases. This result is consistent with the expectation that for stiffer samples and at small indentations, the strain field in Figure S3 is less influenced by the substrate. The χ^2 per degree of freedom improves modestly (by factors of approximately 0.9-1.0) with respect to the semi-infinite case when setting the holdfast height to the measured finite values.

We compute the correlation coefficient between the measured holdfast heights and the fitted elastic moduli; for the 16 h data we find a correlation coefficient of $\rho_{h,E} = -0.20$ and for the 64 hr data $\rho_{h,E} = -0.29$, indicating a weak negative correlation between sample thickness and elastic modulus. We speculate that the weakness of this negative correlation is due to the fact that the holdfast is very stiff, with $E \sim 10^8 - 10^9$ Pa, and therefore the effect of finite sample thickness is not probed at small indentations as strongly it would be for less stiff materials such as typical biological samples, where $E \sim 10^3 - 10^4$ Pa.

Finally, Fig. G-H and Fig. I-J also demonstrate that the finite thickness correction to the Hertz model for the bulk does not capture the transition and surface layer behaviors, necessitating a multi-regime description of the material properties of the holdfast.

4 Young's modulus as a spring constant

To make connection between the fitted results for the holdfast Young's modulus and effective spring constant, we consider holdfast as an elastic bar comprised of a bundle of ideal springs of length h, compressed by δ along the length dimension. The number of springs in parallel is proportional to the cross-sectional area A^* of the bar. Hence, the force applied to each spring is proportional to the total applied force F divided by the cross-sectional area A^* . Hooke's law for each spring in the bundle is

$$\frac{F}{A^*} = E\frac{\delta}{h}$$

where E is Young's modulus. Therefore, $k = EA^*/h$. We take A^* to be an effective cross-sectional area of the tip during indentation, $A^* = \alpha\pi R_{tip}^2$, where $\alpha < 1$. While the indentation is not uniform across the cross-sectional area of the tip (i.e., each spring in the bundle is not compressed by the same amount), we approximate it as constant. For an indentation of $\delta/R_{tip} \sim 2/3$, for a spherical indenter, we have $A^* \sim (5/9)\,A$. Using typical holdfast parameter values $E \sim 0.36 \pm 0.12 \times 10^9$ Pa, $h \sim 30$ nm, $R_{tip} = 15$ nm, and $\alpha \sim 5/9$, we find $k \sim 5 \pm 1.7$ N/m, consistent with the fitted value of $k = 7.1 \pm 0.5$ N/m.

References

[1] Radmacher M. 2007. Studying the mechanics of cellular processes by atomic force microscopy. Cell Mechanics 83:347-372.

[2] Li G, Smith CS, Brun YV, Tang JX. 2005. The Elastic Properties of the Caulobacter crescentus Adhesive Holdfast Are Dependent on Oligomers of N-Acetylglucosamine. Journal of Bacteriology 87:257-265.

[3] Rebelo LM, de Sousa JS, Mendes Filho J, Radmacher M. 2013. Comparison of the viscoelastic properties of cells from different kidney cancer phenotypes measured with atomic force microscopy. Nanotechnology 24:055102.

[4] Guz N, Dokukin M, Kalaparthi V, Sokolov I. 2014. If Cell Mechanics Can Be Described by Elastic Modulus: Study of Different Models and Probes Used in Indentation Experiments. Biophysical Journal 107:564-575.

[5] Dimitriadis EK, Horkay F, Maresca J, Kachar B, Chadwick RS. 2002. Determination of elastic moduli of thin layers of soft material using the atomic force microscope. Biophysical Journal 82:2798-2810.

[6] Bonilla MR, Stokes JR, Gidley MJ, Yakubov GE. 2015. Interpreting atomic force microscopy nanoindentation of hierarchical biological materials using multi-regime analysis. Soft Matter 2015 11:1281-1292.

[7] Butt HJ, Cappella B, Kappl M. 2005. Force measurements with the atomic force microscope: Technique, interpretation and applications. Surface Science Reports 59:1-152.

[8] Camesano TA, Logan BE. 2000. Probing bacterial electrosteric interactions using atomic force microscopy. Environmental Science & Technology 34:3354-3362.

[9] de Gennes PG. 1976. Scaling Theory of Polymer Adsorption. Journal De Physique. 37:1445-1452.

[10] Alexander S. 1977. Adsorption of Chain Molecules with a Polar Head a-Scaling Description. Journal De Physique. 38:983-987.

[11] Isralachvili, J. 2011. Intermolecular and surface force.

[12] Butt HJ, Kappl M, Mueller H, Raiteri R, Meyer W, Rhe J. 1999. Steric forces measured with the atomic force microscope at various temperatures. Langmuir 15:2559-2565.

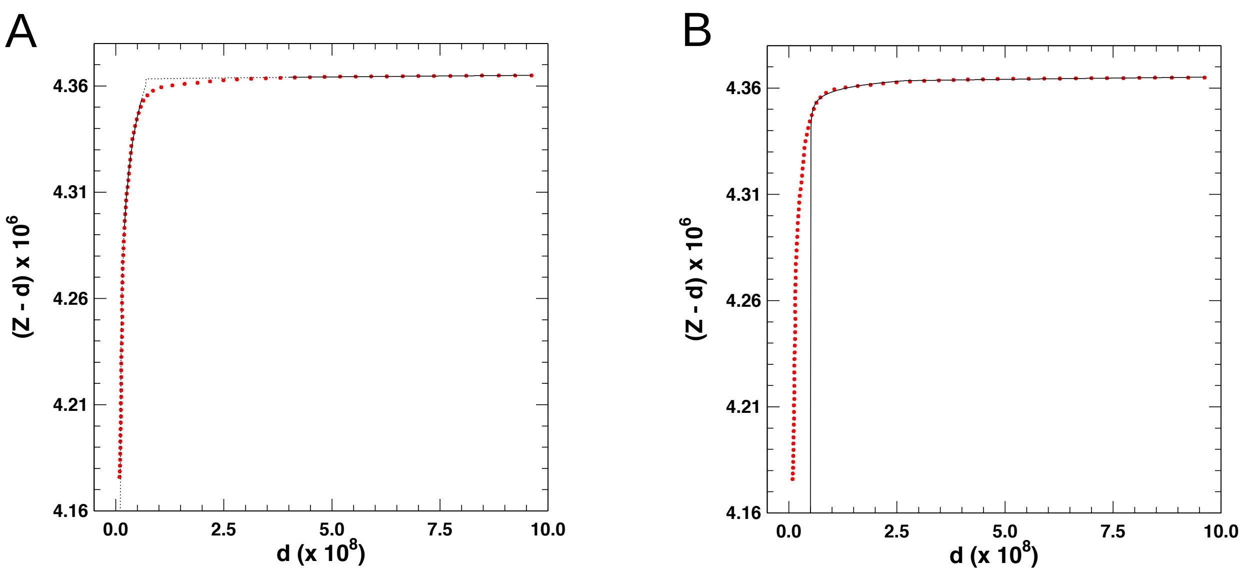

Figure A-B: Hertzian and brush layer fits to a representative 16 h data set (A) excluding the transition region at the surface of the holdfast, with fit parameters given by: $d_0 = 1.15 \times 10^{-9}\,\mathrm{m}, Z_0 = 4.364 \times 10^{-6}\,\mathrm{m}, E = 1.79 \times 10^{9}\,\mathrm{Pa}, L_0 = 2.15 \times 10^{-7}\,\mathrm{m}, \Gamma = 2.37 \times 10^{16}\,\mathrm{m}^{-2}$; and (B) requiring the fits to be continuous at the surface of the holdfast, with fit parameters given by: $d_0 = 5.08 \times 10^{-9}\,\mathrm{m}, Z_0 = 4.367 \times 10^{-6}\,\mathrm{m}, E = 1.09 \times 10^{9}\,\mathrm{Pa}, L_0 = 2.24 \times 10^{-8}\,\mathrm{m}, \Gamma = 1.96 \times 10^{17}\,\mathrm{m}^{-2}$. The axes units are in meters.

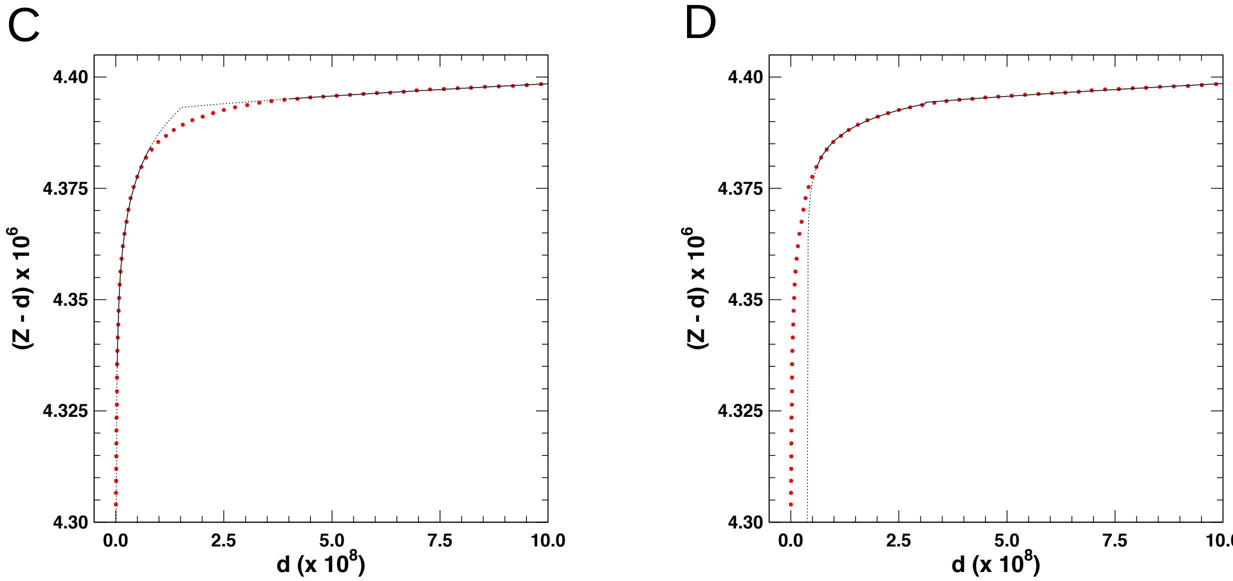

Figure C-D: Hertzian and brush layer fits to a representative 64 h data set (C) excluding the transition region at the surface of the holdfast, with fit parameters given by: $d_0 = 1.95 \times 10^{-10}\,\mathrm{m}, Z_0 = 4.391 \times 10^{-6}\,\mathrm{m}, E = 2.61 \times 10^{8}\,\mathrm{Pa}, L_0 = 7.99 \times 10^{-8}\,\mathrm{m}, \Gamma = 7.54 \times 10^{16}\,\mathrm{m}^{-2}$; and (D) requiring the fits to be continuous at the surface of the holdfast, with fit parameters given by: $d_0 = 3.96 \times 10^{-9}\,\mathrm{m}, Z_0 = 4.395 \times 10^{-6}\,\mathrm{m}, E = 2.64 \times 10^{8}\,\mathrm{Pa}, L_0 = 3.47 \times 10^{-8}\,\mathrm{m}, \Gamma = 1.49 \times 10^{17}\,\mathrm{m}^{-2}$. The axes units are in meters.

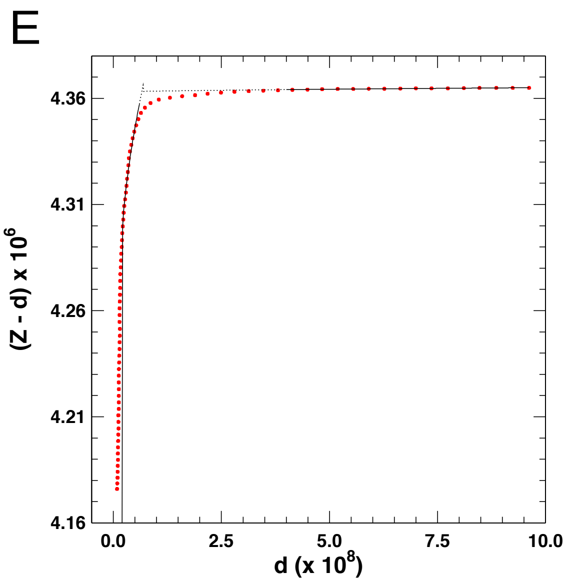

Figure E: Hertzian fits to both the holdfast bulk and surface layer for a representative 16 h data set, excluding the transition region at the surface of the holdfast, with fit parameters given by: $d_0 = 2.03 \times 10^{-9}\,\mathrm{m}, Z_0 = 4.365 \times 10^{-6}\,\mathrm{m}, E_{bulk} = 1.80 \times 10^{9}\,\mathrm{Pa}, E_{layer} = 4.30 \times 10^{5}\,\mathrm{Pa}$. The χ^2 per degree of freedom measuring the goodness of fit is ~ 2 times greater than for the brush layer model. The axes units are in meters.

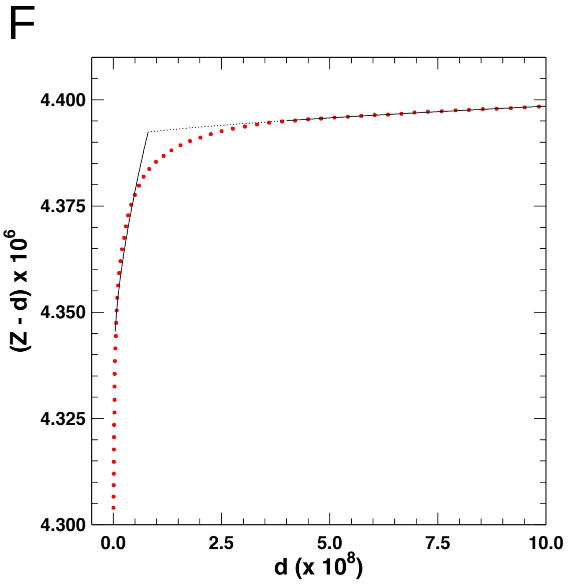

Figure F: Hertzian fits to both the holdfast bulk and surface layer for 64 h data set, excluding the transition region at the surface of the holdfast, with fit parameters given by: $d_0 = 3.47 \times 10^{-10}\,\mathrm{m}, Z_0 = 4.391 \times 10^{-6}\,\mathrm{m}, E_{bulk} = 2.65 \times 10^{8}\,\mathrm{Pa}, E_{layer} = 1.21 \times 10^{6}\mathrm{Pa}$. The χ^2 per degree of freedom measuring the goodness of fit is ~ 120 times greater than for the brush layer model. The axes units are in meters.

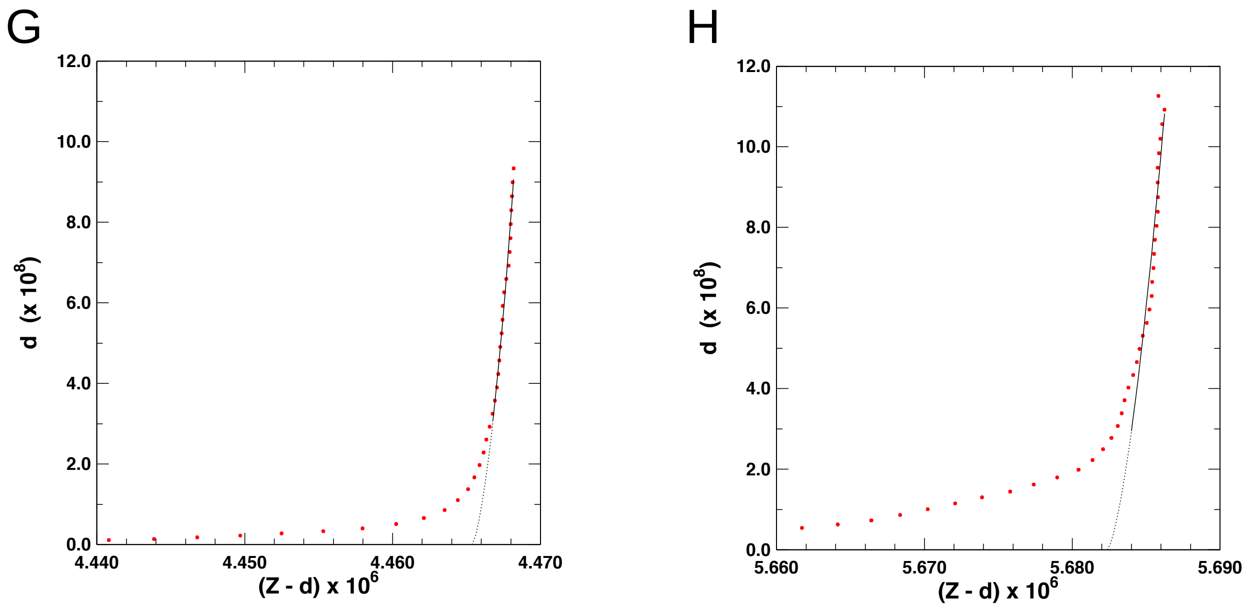

Figure G-H: Hertzian model fits to the holdfast bulk, including correction for finite thickness of the samples for representative samples (16 h) with different heights and approximately equal elastic moduli. The parameter d_0 is set to the value determined from previous fits (brush layer + Hertz for semi-infinite sample), while Z_0 and $E = E^{corr}$ are allowed to float. (G) h is set to the measured height of the sample, $h_{measured} = 25.8\,\mathrm{nm}$, and $E_{h\gg1} = 1.13 \times 10^9$ Pa. We find $E^{corr} = 1.54\,E_{h\gg1}$. (H) h is set to the measured height of the holdfast, $h_{measured} = 81.7\,\mathrm{nm}$, and $E_{h\gg1} = 1.11 \times 10^9$ Pa. We find $E^{corr} = 1.14\,E_{h\gg1}$. We note that the ratio of the elastic modulus with corrections for finite thickness to that for the semi-infinite sample is greater for a thinner sample. The axes units are in meters.

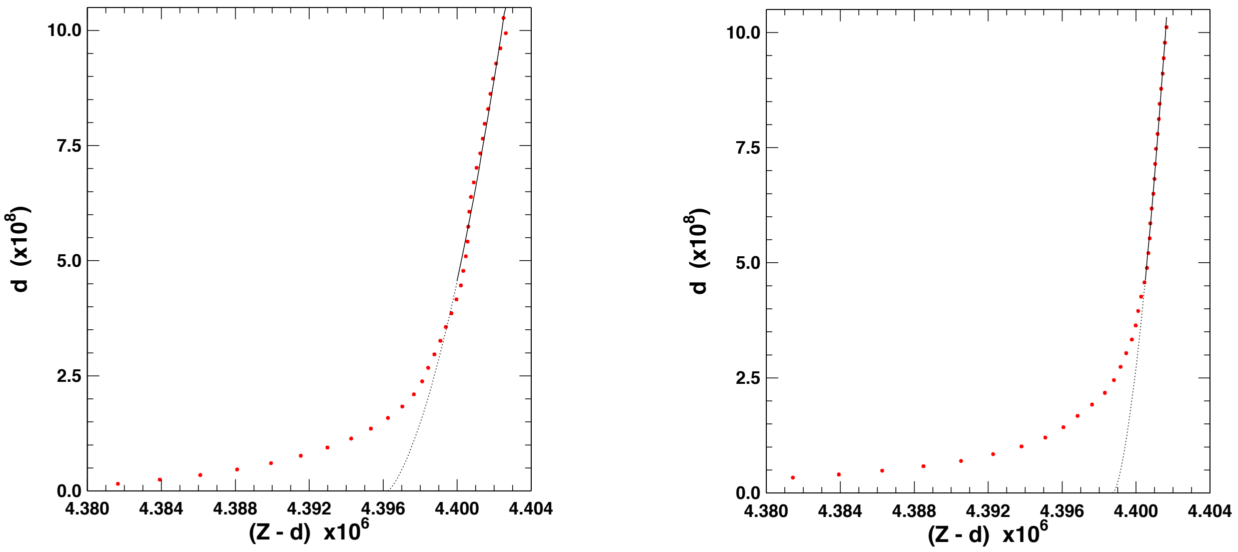

Figure I-J: Hertzian model fits to the holdfast bulk, including correction for finite thickness of the samples for representative samples (64 h) with different elastic moduli and approximately equal heights. The parameter d_0 is set to the value determined from previous fits (brush layer + Hertz for semi-infinite sample), while Z_0 and $E = E^{corr}$ are allowed to float. (I) h is set to the measured height of the sample, $h_{measured} = 34.2\,\mathrm{nm}$, and $E_{h\gg1} = 0.38 \times 10^9$ Pa. We find $E^{corr} = 1.67\,E_{h\gg1}$. (J) h is set to the measured height of the holdfast, $h_{measured} = 33.1\,\mathrm{nm}$, and $E_{h\gg1} = 1.12 \times 10^9$ Pa. We find $E^{corr} = 1.40\,E_{h\gg1}$. We note that the ratio of the elastic modulus with corrections for finite thickness to that for the semi-infinite sample is greater for a softer sample. The axes units are in meters.
